# MIG-21 interacts with Wnt and Netrin signaling in gonad migration in *C. elegans*

**DOI:** 10.1371/journal.pgen.1011866

**Published:** 2025-09-15

**Authors:** Xin Li, Kacy Lynn Gordon

**Affiliations:** 1 Department of Biology, The University of North Carolina at Chapel Hill, Chapel Hill, North Carolina, United States of America; 2 UNC Lineberger Comprehensive Cancer Center, Chapel Hill, North Carolina, United States of America; Centre National de la Recherche Scientifique, FRANCE

## Abstract

The gonad of *Caenorhabditis elegans* hermaphrodites is a longstanding model of cell migration, stem cell niche function, and organogenesis, but it has not yet been investigated using single-cell RNA-sequencing (scRNA-seq). Using a recently published scRNA-seq dataset of adult *C. elegans* hermaphrodites, we identified a previously unknown regulator of the leader cell of gonad migration (the distal tip cell, or DTC). The gene *mig-21* is both highly and specifically expressed in the DTC, yet has no known role in that cell. However, *mig-21* regulates cell migration in other developmental contexts. Using classical genetics techniques, RNAi knockdown, and live cell imaging, we discovered that *mig-21* acts synergistically with the Wnt and Netrin pathways to guide anteroposterior and dorsoventral phases of migration in the DTC at the level of signaling, not DTC cell structure. Known interactors of *mig-21* in other cell types–like PTP-3C–also act with MIG-21 in DTC migration. Despite its expression in stationary adult DTCs, *mig-21* does not play a role in the cessation of DTC migration but instead seems to impart continued sensitivity of the DTC to Wnt and Netrin in adulthood. This study reveals additional complexity of signaling integration between major regulators of germline stem cell niche migration, and as a proof of concept it demonstrates the utility of scRNA-seq datasets in revealing testable hypotheses about genetic networks that were masked by redundancy in traditional screening methods.

## Author summary

Single-cell RNA-sequencing is increasingly used in developmental biology studies. Ideally, these studies, like the genome sequence resources that preceded them, will be useful for reanalysis beyond their initial publication. We used a recently published cell atlas for adult *C. elegans* nematode worms to identify candidate genes that may regulate our cell of interest, the leader cell of gonad formation called the distal tip cell (DTC). The gene which maximizes expression level and specificity for that cell type, *mig-21*, had no known function in the DTC but regulates cell migration in other contexts. We discovered that *mig-21* mutants have low-level DTC migration defects, and that *mig-21* mutation strongly enhances known migration defects caused by loss-of-function in both Wnt and Netrin signaling pathways in the DTC. The known genetic interactions between Wnt and Netrin signaling in the DTC rely on *mig-21.* We situate *mig-21* in the Netrin pathway as an integrator of Wnt with Netrin signaling. Our study not only reveals new regulatory complexity in gonad organogenesis, but demonstrates the utility of single-cell RNA-sequencing datasets for generating biologically meaningful hypotheses. We suggest that these datasets may be especially useful in identifying genes with roles that have been masked by redundancy in functional genetic screens.

## Introduction

Single-cell RNA sequencing (scRNA-seq) approaches chemically label cDNA made from transcripts from single cells before sequencing and then identify cell types by transcriptome similarity via clustering algorithm [1]. scRNA-seq has rocketed to prominence in studies of developmental biology in the past decade [2]. Cell atlases constructed by single-cell RNA-sequencing are being generated for model organisms and human organs across a range of genetic and physiological states, enabling for example the discovery of rare cell populations with importance in human disease [3]. Ideally, scRNA-seq datasets will lead to new biology being explored well beyond their initial publications. Like genome sequences, these datasets could become precious tools not only for further bioinformatic analyses, but for hypothesis generation for functional genetics studies.

We used a recently published *C. elegans* scRNA-seq dataset [4] to study a cell of interest: the *C. elegans* hermaphrodite gonad stem cell niche, called the distal tip cell (DTC). Each of the two gonad arms of the *C. elegans* hermaphrodite has a single DTC. The DTC is a migratory germline stem cell niche that has long served as a model for cell migration and stem cell niche biology [5,6]. Its stereotyped migration during post-embryonic development patterns the correct U-shaped gonadal morphology of each adult gonad arm. In the L2 and early L3 larval stages, the DTCs migrate away from each other along the ventral body wall in an anterior or posterior direction. In the L3 larval stage, each DTC makes a 90-degree turn off the ventral body wall, crosses the lateral epidermis, and then makes another 90-degree turn onto the dorsal body wall to face the midbody. These turns pattern the bend region of the mature gonad. During the L4 larval stage, each DTC migrates along the dorsal body wall and comes to rest at the dorsal midbody [5].

The genetics of DTC migration and cessation have been studied for decades. DTC migration is governed by two major signaling pathways: Netrin signaling that primarily governs D/V migration (the first turn) and Wnt signaling that primarily governs A/P migration (the second turn) [7]. Proper A/P and D/V guidance of the DTC results from the integration of inputs from both networks, and they demonstrate some degree of redundancy [8]. During reproductive adulthood, the DTC is stationary, highly elaborated, and continues to signal to the underlying germ stem cells to maintain them in an undifferentiated state. We asked if previously unknown regulators of the DTC could be identified by examining highly expressed genes detected in that cell type by scRNA-seq.

We used the web app WormSeq.org [9] for the dataset reported by [4] to look for DTC “marker genes”. Surprisingly, the gene with the highest “marker score” is *mig-21*, which has been studied in the context of neuronal cell migration but was not known to function in the DTC. *mig-21* was initially identified in a screen for genes that affect touch receptor neuron development [10], many of which proved to be essential for proper anterior-posterior migration of the progenitors of these cells. Like UNC-5, MIG-21 is a thrombospondin repeat (TSPI)-containing transmembrane protein that interacts with the Netrin receptor UNC-40/DCC to regulate cell polarization and migration of Q neuroblasts during early larval development [11]. Like the two DTCs, the two Q neuroblasts initially migrate away from one another along the A/P axis [12]. MIG-21-dependent polarization of QL and QR restricts the threshold response to the EGL-20/Wnt gradient in these cells [11]. The role of *mig-21* in Q neuroblast migration in response to the same Wnt and Netrin signals that guide DTC migration makes it a plausible candidate to examine in the DTC.

In this study, we established a role for *mig-21* in DTC migration, focusing on the Wnt and Netrin signaling pathways that regulate both Q neuroblast and DTC migration. We also examined whether known interactors of *mig-21* in Q neuroblasts coregulate DTC migration. Finally, we examined whether a known regulator of DTC migration cessation interacts with *mig-21.* This work provides new insights into the complex interplay of signaling pathways that guide DTC migration and highlights the important role of *mig-21*, emphasizing the power of scRNA-seq in hypothesis generation for probing gene function in development.

## Results and discussion

### DTC migration is regulated by *mig-21*

**Fig 1:**
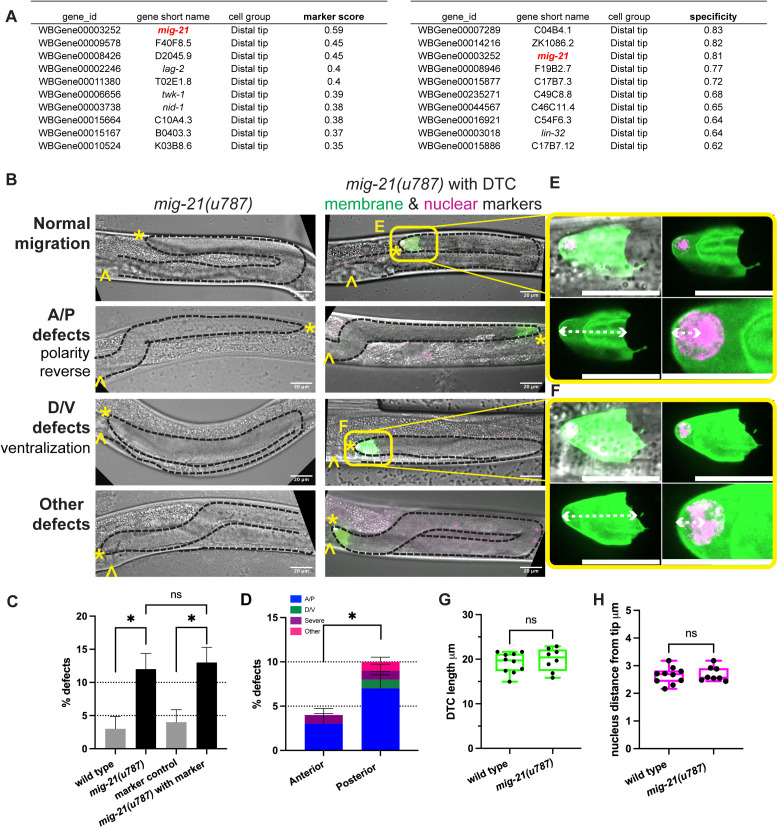
*mig-21* regulates DTC migration but not cell structure. (A) Top 10 genes ranked by marker score (left) and specificity (right) from WormSeq.org [9] for the distal tip cell at the young adult stage. *mig-21* is highlighted as the only gene on both lists. (B) Micrographs on the left: DIC imaging of *C. elegans* hermaphrodites at the late larval L4 stage for *mig-21(u787)* worms. Micrographs on the right: DIC merged with fluorescence imaging of the same stage *mig-21(u787)* strain bearing a transgene that marks the membrane of the DTC, *cpIs122[lag-2p::mNeonGreen:: PLC*^*δ*^^*PH*^], and a nuclear marker inserted at the endogenous *lag-2* locus *lag-2(bmd202[lag-2::P2A::H2B::mT2])* [16]. Images are Z-projections through the thickness of the gonad required to capture the whole distal gonad. Black dashed lines outline gonads. Anterior left and ventral down. Yellow asterisks mark DTC; yellow carets mark the proximal vulval position. Scale bar: 20 μm. Yellow boxes indicate the positions of insets shown in E and F. (C) Percentage of DTC migration defects across experimental groups. Low-penetrance but significant defects were identified in *mig-21(u787)* alone and with fluorescence markers. Wild type N2 strain control (n=3/92), *mig-21(u787)* (n=23/189), p < 0.05; markers control (n=4/104), *mig-21(u787)* with markers (n=28/215), p < 0.05. Introducing fluorescence markers did not significantly alter *mig-21(u787)* defect rates (p > 0.05). (D) Percentage of worms with migration defects in anterior vs. posterior gonad arms of *mig-21(u787)* worms. Migration defects were more frequent in the posterior gonad arm (n=18/189) compared to the anterior arm (n=7/189), p < 0.05. Anterior-posterior polarity (A/P) defects (n=19/189) were significantly more common than dorsal-ventral polarity (D/V) defects (n=1/189), p < 0.0001. (C-D) All sample sizes refer to individual worms. Error bars represent the standard error of the sample proportion. Statistical analysis was performed using a pairwise proportion test, with p-values adjusted for multiple comparisons via the Benjamini-Hochberg procedure. Significant differences are indicated between groups where applicable. ****p < 0.0001; ***p < 0.001; **p < 0.01; *p < 0.05; ns = not significant. The corresponding sample sizes and statistics are presented in Tables A–D in S1 File; additional details like raw data collections, calculated means, and standard errors of the mean (SEM) are presented in Sheets A-D in S2 File. (E-F) Enlargement of distal tip fluorescence images from 1B, showing normal (E) and defective (F) migration. Annotations show measurements quantifying DTC morphology and nuclear localization (graphed in 1G-H). White dashed lines represent measurement parameters: (Bottom panels Left) DTC length, as the linear distance from the distal tip to the proximal boundary of the DTC; (Bottom panels Right) nuclear position, as the distance from the distal tip to the geometric center of the nucleus. Scale bars 20 μm except bottom right panels 10 μm. (G) Box plots overlaid with all datapoints measuring the DTC length. Sample sizes refer to individual gonads. Wild type N2 control n=10, and *mig-21(u787)* n=8. Statistical significance was calculated by unpaired, two-tailed Student’s t-tests, error bars represent ±SEM. No significant difference was observed, t=0.6080, df=16, 95% confidence interval -1.737 to 3.135 μm, p = 0.7374. (H) Box plots overlaid with all datapoints measuring the DTC nuclei position. Sample sizes refer to individual gonads. Wild type N2 control n=10 and *mig-21(u787)* n=8. Statistical significance was calculated by unpaired, two-tailed Student’s t-tests, error bars represent ±SEM. No significant difference was observed, t=0.4361, df=16, 95% confidence interval -0.2280 to 0.3461 μm, p = 0.7531.

We identified *mig-21* as our gene of interest by looking for DTC-expressed genes with a high “marker score” (genes with relatively high expression levels and relatively specific expression) and “specificity score” (determined by Jensen-Shannon distance) [13] in scRNAseq results [4]. The gene *mig-21* is at the top of the “marker” list and is third for “specificity” in the DTC (Fig 1A). It is the only gene that appears in the top ten genes of both sorts.

First, we examined the effect of *mig-21* loss-of-function on DTC migration by analyzing a strain bearing the *mig-21(u787)* mutant allele which has a premature amber stop codon in the extracellular domain and is considered to be a putative null [11]. Amber stop codons are not read-through in *C. elegans* [14]. The mutant gene may produce a 64 amino acid (potentially secreted) protein fragment lacking thrombospondin, transmembrane, and intracellular domains, or more likely it may be subject to nonsense-mediated decay, which is a global eukaryotic system for eliminating transcripts with premature stop codons [15].

In *mig-21(u787)* mutants, we observed a low penetrance defect in DTC migration in which some DTCs failed to execute the two turns correctly (Figs 1B, 1C and S1A). We observe low-penetrance DTC migration defects when we treat both wild-type worms and worms with DTC-specific RNAi activity (see Methods) with *mig-21* RNAi (S1B Fig). Furthermore, *mig-21* RNAi treatment does not enhance the *mig-21(u787)* mutant phenotype, suggesting that *mig-21(u787)* causes a cell-autonomous loss-of-function phenotype in the DTC (S1B Fig).

We categorized defects into anterior/posterior (A/P) vs. dorsal/ventral (D/V) polarity defects according to the specific phase of migration that was affected, as well as “other” migration defects and “severe” defects in which gonad growth failed or formed a disorganized mass (Figs 1B and S1A). *mig-21(u787)* mutants exhibited a significantly higher proportion of A/P defects than D/V defects, with more defects observed in the posterior gonad arm than in the anterior (Fig 1D).

DTC migration defects are caused by signaling defects [8], as well as by cell structural abnormalities including those that arise from defects in Rac GTPase signaling [16,17] or actomyosin-based contractility and nuclear mispositioning [18]. To further investigate the potential role of *mig-21* in influencing DTC shape or nuclear position during migration, we crossed *mig-21* mutants with a DTC marker strain that has membrane-localized DTC fluorescence and a nuclear histone tag *(lag-2p::mNeonGreen::PLC*^*δ*^^*PH*^*; lag-2(bmd202[lag-2::P2A::H2B::mT2])* [16]. The presence of these transgenes did not enhance DTC migration defects in either a wild-type or *mig-21(u787)* background (Fig 1C). Defects caused by knockdown of Rac-family GTPase genes *mig-2/*RhoG and *ced-10*/Rac were not significantly enhanced by *mig-21(u787)* after correcting for multiple comparisons (S1C-E Fig and Table AP–AR in S1 File), perhaps because these Rac-type GTPases are sometimes (but not always) functionally redundant [16,19].

In the L4 stage, both control and *mig-21(u787)* mutant DTCs maintained their normal shape and displayed well-defined nuclei polarized in the direction of migration (Fig 1E–1H), suggesting that *mig-21* likely interacts with signaling pathways involved in DTC guidance rather than cellular integrity or nuclear positioning, with a specific impact on anterior-posterior directional polarity, especially in the posterior gonad arm.

### *mig-21* interacts genetically with Wnt signaling in the DTC

Wnt signaling is the main regulator of DTC and Q neuroblast migration along the A/P axis, the latter dependent on *mig-21* [12]. Redundancy among Wnt pathway members and between Wnt and Netrin signaling had previously been described by revealing epistasis tests [8,19]. Genetic redundancy revealed by superadditive/synergistic interaction does not suggest true molecular redundancy between Wnt and Netrin pathway members, but redundancy in the guidance information they impart. We hypothesized that *mig-21* may also function redundantly in this sense (which from now on we will refer to as synergistically) with Wnt signaling to guide A/P migration, explaining why it had not previously been discovered as a regulator of DTC migration.

To assess the genetic interaction between *mig-21* and the Wnt signaling pathway, RNAi by feeding was used to knock down Wnt pathway genes in the wildtype and *mig-21(u787)* backgrounds. We targeted *mom-5* (which encodes the main Frizzled receptor that acts during DTC migration to guide A/P polarity [19]), *lin-17* (another Frizzled [8]), and the five Wnt ligand genes, *egl-20* (which forms a Wnt gradient crucial for Q neuroblast migration [20]), *lin-44*, *mom-2, cwn-1, and cwn-2*. We predicted that if *mig-21* acts synergistically to the Wnt signaling pathway, we will see an enhancement of Wnt knockdown phenotypes in *mig-21(u787)* mutants.

**Fig 2:**
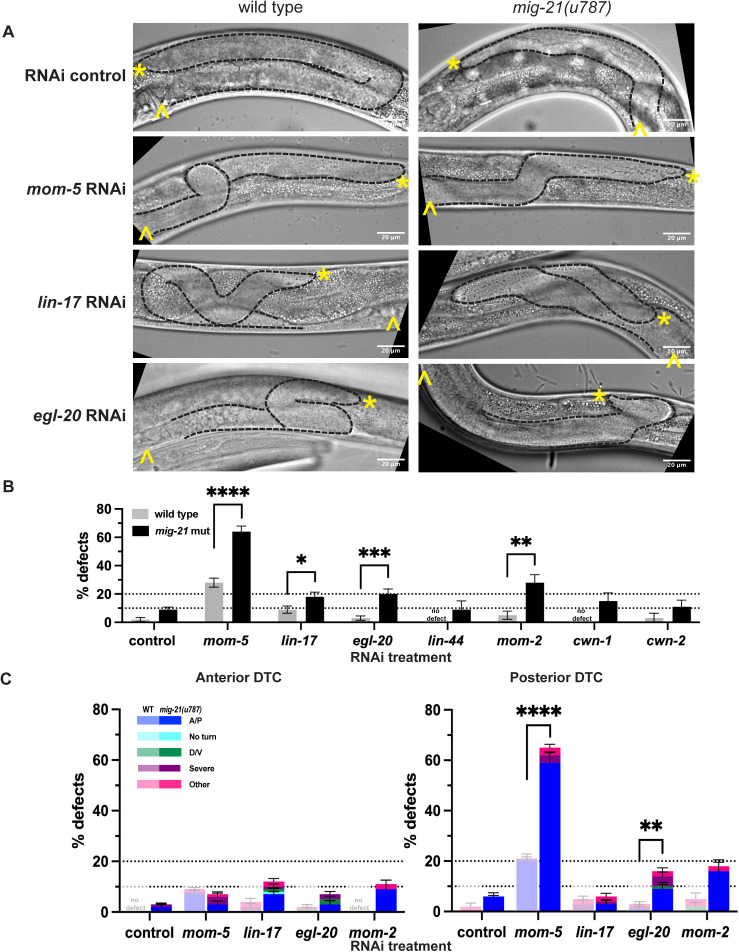
*mig-21* regulates Wnt signaling in the DTC. (A) Micrographs: DIC imaging of *C. elegans* hermaphrodites at the late larval L4 stage, comparing wild type N2 (left) and *mig-21(u787)* (right) under RNAi control L4440 empty vector, *mom-5* RNAi, *lin-17* RNAi, or *egl-20* RNAi feeding treatment to assess DTC migration defect phenotypes. Images are Z-projections through 2-3 μm showing the distal gonad. Anterior left and ventral down. Black dashed lines outline gonads. Yellow asterisks mark DTC; yellow carets mark the proximal vulval position. Scale bar: 20 μm. (B) All DTC migration defects across experimental groups, including wild type N2 (gray) and *mig-21(u787)* (black) strains, under RNAi control L4440 empty vector (wild type n=102; *mig-21(u787)* n=253), Frizzled receptors *mom-5*(wild type=202; *mig-21(u787)* n=150) and *lin-17*(wild type n=125; *mig-21(u787)* n=138) and Wnt ligands *egl-20* (wild type n=110; *mig-21(u787)* n=131)*, lin-44* (wild type n=20; *mig-21(u787)* n=22)*, mom-2*(wild type n=58; *mig-21(u787)* n=64)*, cwn-1* (wild type n=23; *mig-21(u787)* n=39)*, cwn-2* (wild type n=29; *mig-21(u787)* n=45) RNAi feeding treatments. Significant enhancements of migration defects by *mig-21(u787)* were observed in *mom-5*, *lin-17*, *egl-20*, and *mom-2* groups. (C) DTC migration defects for the groups with significant enhancement by *mig-21(u787)* in anterior (left) and posterior (right) arms. Lighter one means wild type groups, darker one means *mig-21(u787)* groups. (B-C) All sample sizes refer to individual worms. On the graphs, “no defect” means no defect observed in that group. Error bars represent the standard error of the sample proportion. Statistical analysis was performed using a pairwise proportion test, with p-values adjusted for multiple comparisons via the Benjamini-Hochberg procedure. Significant differences are indicated between groups where applicable. ****p < 0.0001; ***p < 0.001; **p < 0.01; *p < 0.05; no mark means the comparison was not statistically significant. The corresponding sample sizes and statistics are presented in Tables E–J in S1 File; additional details like raw data collections, calculated means, and SEM are presented in Sheets E-F in S2 File.

After empty-vector control RNAi treatment, *mig-21(u787)* mutants showed the minor A/P migration defect rate (n = 23/253, 9%) that we observed on standard growth media. Knockdown of *mom-5*/Frizzled with RNAi in wild-type worms caused a 28% total per worm A/P migration defect (n = 56/202, Fig 2A and 2B), which agrees with previous *mom-5* RNAi results [16,19]. In the *mig-21(u787)* background, the incidence of A/P migration defects after *mom-5* RNAi increases to 64% (n = 96/150, Fig 2A and 2B), which our statistical model confirms is a superadditive, synergistic, interaction (p < 0.00001). This enhancement has an almost complete bias for the posterior DTC (Fig 2C). Incidence of A/P migration defects caused by *lin-17* RNAi treatment of wild-type worms was more modest (n = 11/125, 9%), but was also enhanced over twofold by *mig-21(u787)* (n = 25/138, 18%) with the opposite bias, for the anterior DTC (Fig 2C); this enhancement suggests an additive effect. We thus conclude that *mig-21* functions synergistically with the MOM-5/Frizzled Wnt receptor during DTC migration, specifically in the posterior DTC.

A Wnt ligand-independent function of *lin-17*/Frizzled has recently been discovered in the earliest asymmetries in the somatic gonad [21], however, DTC migration is Wnt-dependent [8]. We next tested genetic interactions of Wnt ligand genes with *mig-21*. When they have been investigated previously, single loss of function of the five Wnt ligand genes caused negligible DTC migration defects [8]. Indeed, we did not observe major A/P migration defects after wild-type worms were exposed to RNAi for any of the Wnt ligand genes. The *mig-21(u787)* mutation significantly enhanced migration defects of *egl-20*/Wnt and *mom-2*/Wnt RNAi. We conclude that *mig-21* works synergistically with liganded Frizzled receptors (primarily MOM-5) to guide A/P polarity of (primarily posterior) DTC migration, with special sensitivity for EGL-20, the Wnt ligand to which *mig-21* regulates the response in the Q neuroblasts [11].

### *mig-21* interactors known from the Q neuroblast also regulate DTC migration

**Fig 3:**
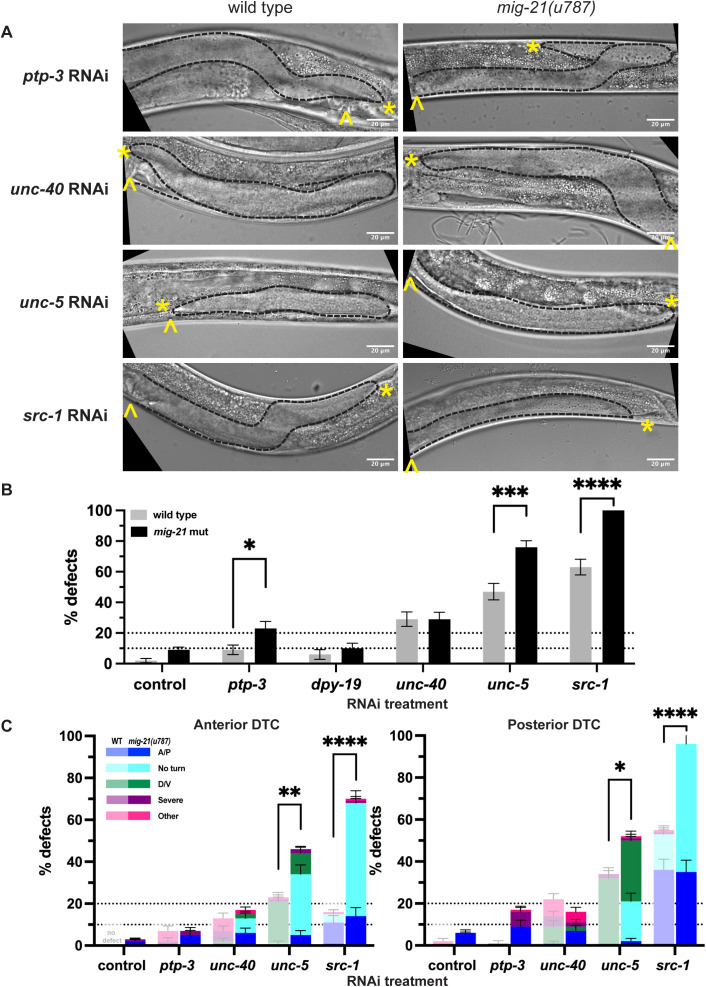
*mig-21* works with *ptp-3* and regulates Netrin signaling in the DTC. (A) Micrographs: DIC imaging of *C. elegans* hermaphrodites at the late larval L4 stage, comparing wild type N2 (left) and *mig-21(u787)* (right) under RNAi control L4440 empty vector, LAR receptor *ptp-3*, C-mannosyltransferase *dpy-19* and Netrin receptors *unc-40, unc-5*, or *src-1* RNAi feeding treatment. Images are Z-projections through 2-3 μm showing the distal gonad. Anterior left and ventral down. Black dashed lines outline gonads. Yellow asterisks mark DTC; yellow carets mark the proximal vulval position. Scale bar: 20 μm. (B) All DTC migration defects across experimental groups, including wild type N2 (gray) and *mig-21(u787)* (black) strains, under RNAi control L4440 empty vector, *ptp-3* RNAi (wild type=81; *mig-21(u787)* n=87), *dpy-19* RNAi (wild type=53; *mig-21(u787)* n=79), *unc-40* RNAi (wild type=93; *mig-21(u787)* n=101), *unc-5* RNAi(wild type=85; *mig-21(u787)* n=103), or *src-1*(Wild type=89; *mig-21(u787)* n=72) RNAi feeding treatment. (C) DTC migration defects across different experimental groups in anterior (left) and posterior (right) arms. Lighter one means wild type groups, darker one means *mig-21(u787)* groups.Significant enhancement of the overall defect rate was observed in anterior (p < 0.01) and posterior (p < 0.05) arms of *unc-5* group, with a highly significant increase in the “no turn” phenotype in both the anterior (p < 0.0001) and posterior (p < 0.0001) arms of *unc-5* group. (B-C) All sample sizes refer to individual worms. (B-C) Datasets for wild type strain on L4440 control RNAi vector and *mig-21(u787)* strain on L4440 control RNAi vector are the same as shown in Fig 2B; these controls were pooled across replicates of all RNAi experiments. On the graphs, “no defect” means no defect observed in that group. Error bars represent the standard error of the sample proportion. Statistical analysis was performed using a pairwise proportion test, with p-values adjusted for multiple comparisons via the Benjamini-Hochberg procedure. Significant differences are indicated between groups where applicable. ****p < 0.0001; ***p < 0.001; **p < 0.01; *p < 0.05. The corresponding sample sizes and statistics are presented in Tables K–S in S1 File; additional details like raw data collections, calculated means, and SEM are presented in Sheets G-I in S2 File.

In the Q neuroblasts, MIG-21 partners with PTP-3, which encodes a type IIa receptor phosphotyrosine-phosphatase (RPTP), similar to LAR in some of its isoforms [20], to regulate Wnt-responsive cell polarization. *ptp-3* is not known to function in the DTC, though its transcripts are detectable in the adult [4,9] and L2 DTC [9,22]. RNAi knockdown of *ptp-3* in wild-type worms caused minor incidence of gonad migration defects (n = 7/81, 9% total, Fig 3A and 3B). However, *mig-21(u787)* mutants treated with *ptp-3* RNAi had a total gonad defect rate of 23% (n = 20/87), and enhanced A/P migration defects in the posterior gonad from 1% (n = 1/81) in the control worms on *ptp-3* RNAi to 17% (15/87) in the *mig-21(u787)* mutants on *ptp-3* RNAi, which suggests a synergistic interaction (p = 0.0324, Fig 3C). The *ptp-3(mu245)* allele that causes defects in AQR neuroblast migration [12] does not cause DTC migration defects (S3A and S3B Fig). This allele encodes a premature stop that affects PTP-3A and PTP-3B isoforms, but not PTP-3C, which shares the C-terminal structure with these other isoforms encoded by the portion of the gene targeted by our RNAi clone (see Methods: RNAi and S3C Fig). We thus infer that PTP-3C is the isoform that most likely regulates DTC migration.

Several common factors both regulate the DTC and interact with type IIa RPTPs, including LAR. The guanine nucleotide exchange factor Trio interacts with LAR in mammalian cells [23]; in *C. elegans unc-73*/Trio loss of function phenocopies Rac GTPase loss of function in the DTC [16]. Substrates of LAR, like Beta-catenin and cadherin, are known as regulators of the post-migratory DTC [24,25]. Finally, the LAR receptor mediates adhesion between germline stem cells and the *Drosophila* male germline stem cell niche [26]. Future work on the role of *mig-21/ptp-3* interaction in the DTC is warranted.

Another interactor of *mig-21* in Q neuroblast migration, *dpy-19,* encodes a C-mannosyltransferase [27]. RNAi knockdown of the *dpy-19* gene alone or in combination with *mig-21(u787)* has the same minor DTC migration defect as *mig-21(u787)* alone.

Finally, the Netrin receptor UNC-40/DCC interacts with MIG-21/PTP-3 negatively in QR and acts in parallel in QL. We find that wild-type worms on *unc-40* RNAi have a 29% (n = 27/93) overall DTC migration defect (Fig 3A–3C). When *mig-21(u787)* mutants are put on *unc-40* RNAi, the overall migration defect penetrance does not change (n = 29/101, 29%) or differ between anterior and posterior DTCs, but the nature of the phenotypes changes to include failure to turn at all, and other A/P polarity defects in both the anterior and posterior DTC (n = 19/101, 19%) which are almost never observed (n = 1/93, 1%) after *unc-40* knockdown in wild-type worms (indicating a synergistic interaction when it comes to A/P polarity defects specifically, p = 0.039, Fig 3C). Our results suggest that *mig-21* and *unc-40* have complex and potentially differing interactions in the two DTCs, as they do in the two Q neuroblasts. However, UNC-40 is not the only Netrin receptor in the DTC.

### *mig-21* interacts genetically with the Netrin pathway during DTC migration

The Netrin signaling ligand UNC-6 is produced by the ventral nerve cord and signals via both local and long-range mechanisms, both of which are important for proper neuronal growth cone migration/stabilization [28,29]. This guidance function is achieved through interaction with its receptors, UNC-40/DCC [30], and the Netrin receptor UNC-5 [31]. Netrin signaling confers the dominant D/V polarity information in DTC migration, and UNC-5 regulates D/V DTC migration both independently and redundantly with UNC-40 in transducing a repulsive ventral UNC-6/Netrin signal [32], and in a Netrin-independent manner [17]. Since *mig-21* alters the nature of *unc-40* RNAi defects in the DTC (Fig 3C), we hypothesized *mig-21* may interact with UNC-5/Netrin receptor during DTC migration as well.

Loss of *unc-5*/Netrin receptor function causes ventralization of DTC migration in which the DTC migrates out and back along the ventral body, never crossing to the dorsal body wall [30]. The *mig-21(u787)* allele alone rarely shows evidence of D/V migration defect (<5%, Figs 1D and 3C). Treatment of wild-type worms with *unc-5* RNAi causes a 47% (n = 40/85) overall ventralization defect (Fig 3A) (with a slight posterior bias, Fig 3C), which is in line with previous observations [8,16]. Treatment of *mig-21(u787)* mutants with *unc-5* RNAi synergistically enhanced per-worm D/V migration defects to over 70% (p < 0.0005, n = 73/103). Notably, over 20% of affected gonads in both the anterior and posterior failed to turn (Fig 3C). The “no turn” phenotype is considered to result from defects of both A/P and D/V polarity of the DTC, with the DTC failing to cross to the dorsal side and then failing to turn back towards the midbody [8].

We also tested a known effector of UNC-5 in DTC migration, the non-receptor tyrosine kinase SRC-1/SFK which is recruited by the intracellular domain of UNC-5 and likely leads to cytoskeletal rearrangement in support of proper polarization of migration [18]. Wild-type worms on *src-1* RNAi have A/P polarity defects (including “no turn” defects), as has been reported previously [18,33], primarily in the posterior gonad (n = 47/89, 53% posterior defect rate Fig 3). The *mig-21(u787)* mutant significantly enhances *src-1* RNAi defect rate overall (n = 72/72, 100%; both significantly different Table AS–AX in S1 File, and synergistic p < 0.00005), and especially increases the frequency of “no turn” defects in both the anterior and posterior gonads (anterior n = 39/72, 54% up from 4% in *src-1* RNAi alone; posterior n = 44/72, 61% up from 17% in *scr-1* RNAi alone, Fig 3.) We found that *src-1* RNAi also enhanced the DTC migration defects of the C-terminal deletion allele of *unc-5(e152)* both on its own and with *mig-21(u787)* (to 100% per animal, S3D–S3G Fig and see discussion of *unc-5(e152)* below). While the truncation of *unc-5(e152)* removes most of the UNC-5 C-terminal intracellular domain, it spares the phosphosite tyrosine Y482 [34,35]. Either this site or another part of the remaining intracellular domain retains the ability of the *unc-5(e152)* protein to recruit SRC-1, or SRC-1 has UNC-5-independent function regulating DTC migration, or both.

We thus conclude that *mig-21(u787)* enhances the frequency and severity of migration defects caused by the loss of function of Netrin pathway genes *unc-5* and *src-1*, and likely acts in the same pathway, especially in its contribution to A/P polarity.

### *mig-21* interacts with the Wnt and Netrin receptors cell autonomously to regulate DTC migration

**Fig 4:**
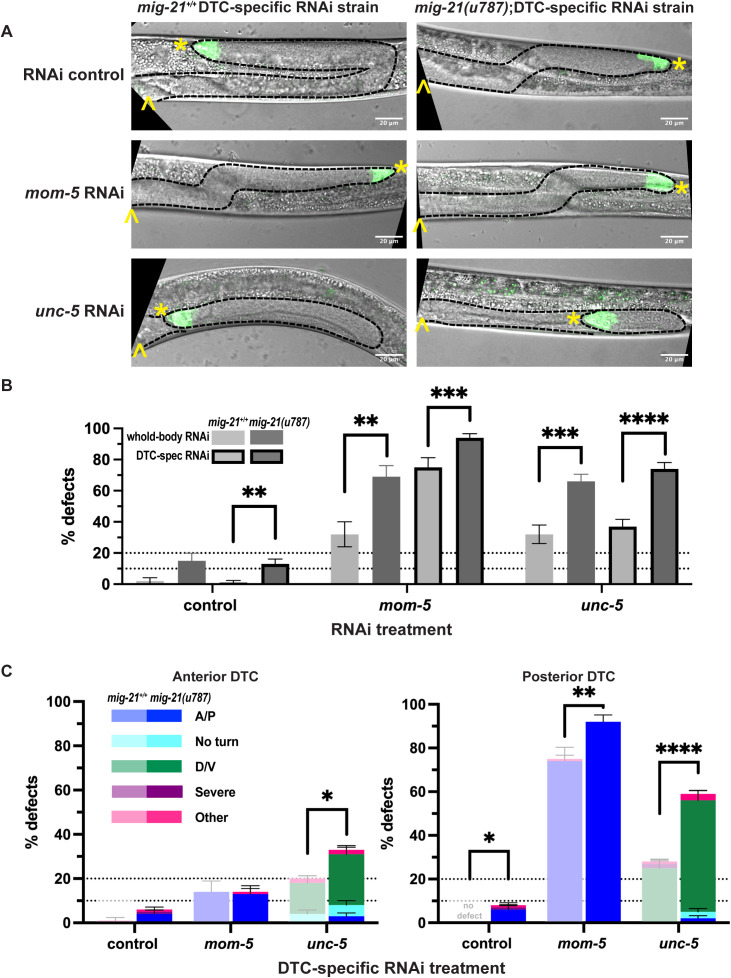
*mig-21* interacts with the Wnt and Netrin receptors cell autonomously to regulate DTC migration. (A) Micrographs: DIC merged with fluorescence DTC imaging of *C. elegans* hermaphrodites at the late larval L4 stage, comparing DTC-specific RNAi strains that carry *mig-21*^*+/+*^ (NK2115 [51], left) vs. the *mig-21(u787)* allele (KLG060, right). Top, RNAi control L4440 empty vector; middle, *mom-5* RNAi; bottom, *unc-5* RNAi feeding treatment. Images are Z-projections through 2-3 μm showing the distal gonad. Anterior left and ventral down. Black dashed lines outline gonads. Yellow asterisks mark DTC; yellow carets mark the proximal vulval position. Scale bar: 20 μm. (B) All DTC migration defects across experimental groups, including wild type N2 (light gray), *mig-21(u787)* (dark gray), *mig-21*^*+/+*^ DTC-specific RNAi strain (bordered light gray), and *mig-21(u787)* DTC-specific RNAi strain (bordered dark gray), under RNAi control L4440 empty vector (wild type n= 1/46; *mig-21(u787)* n= 8/53; NK2115 n= 1/84, and KLG060 n= 17/134); *mom-5* (wild type n= 11/34; *mig-21(u787)* n= 29/42; NK2115 n= 55/73, and KLG060 n= 101/107); and *unc-5* (wild type n= 120/62; *mig-21(u787)* n= 71/108; NK2115 n= 40/108, and KLG060 n= 86/117) RNAi feeding treatment. (C) DTC migration defects across different DTC-specific RNAi treatment experimental groups in anterior (left) and posterior (right) gonad arms. Lighter one means *mig-21*+ groups, darker one means *mig-21(u787)* groups. Significant enhancement of the overall defect rate was observed in posterior (p < 0.01) arms of *mom-5* group; both anterior (p < 0.05) and posterior (p < 0.0001) arms of *unc-5* group. (B-C) All sample sizes refer to individual worms. On the graphs, “no defect” means no defect observed in that group. Error bars represent the standard error of the sample proportion. Statistical analysis was performed using a pairwise proportion test, with p-values adjusted for multiple comparisons via the Benjamini-Hochberg procedure. Significant differences are indicated between groups where applicable. ****p < 0.0001; ***p < 0.001; **p < 0.01; *p < 0.05. The corresponding sample sizes and statistics are presented in Table T–AA in S1 File; additional details like raw data collections, calculated means, and SEM are presented in Sheet J in S2 File.

We next tested the site of action of Wnt and Netrin pathway receptor activity interaction with *mig-21* in regulating DTC migration. As a control, we used the validated DTC-specific RNAi strain background that we used to test for the cell-autonomous role of *mig-21* in DTC migration (S1B Fig). First, as expected, both *mom-5* and *unc-5* RNAi cause DTC migration defects when knocked down specifically in the DTC (Fig 4A and 4B, light gray bars with dark outline). We then introduced the *mig-21(u787)* allele into the DTC-specific RNAi background. On empty vector control RNAi, this strain had a low-level, predominantly A/P migration defect, while the DTC-specific RNAi strain without the mutation did not (Fig 4B and 4C, heavy outline datasets), as expected.

When we compared DTC-specific RNAi treatments to whole-body RNAi for *mom-5* of both *mig-21*^+*/*+^ and *mig-21(u787)* worms, the defect rate was greater in the DTC-specific RNAi strain (compare dark gray and light gray with and without heavy outlines, for both *mig-21*^+*/*+^ and *mig-21(u787)* p.adj < 0.001). We attribute this to the cell-autonomous role of *mom-5* in DTC migration, along with the RNAi-sensitizing *rrf-3(pk1426)* mutation in these strains. The defect rates of *unc-5* RNAi in the whole-body and DTC-specific strains were not significantly different. The *mig-21(u787)* allele enhanced the *mom-5* DTC-specific RNAi A/P polarity defect both overall and in the posterior DTC (Fig 4B and 4C). The *mig-21(u787)* allele significantly enhanced the migration defect caused by DTC-specific *unc-5* loss-of-function overall, and significantly enhanced the ventralization of migration in both the anterior and posterior gonads (Fig 4B and 4C).

These results confirm the cell-autonomous role of MOM-5/Frizzled and the Netrin receptor UNC-5 in guiding DTC directional migration, with *mom-5* being especially important for posterior DTC migration (Fig 4C), which concords with what was previously known about their regulation in the DTC. Transcriptional reporters of *unc-5* [7,36,37] and *mom-5* [19] are expressed in the DTC from the time of the first turn. Recent scRNA-seq databases indicate that both genes are expressed in the DTC in the L4 stage [38] as well as in one-day adults, less than a day after migration ends [4,39].

### *mig-21* is required for *unc-5* genetic interactions with the Wnt pathway

Netrin pathway signaling confers the dominant D/V polarity information, and Wnt pathway signaling confers the dominant A/P polarity information during DTC migration, however, the two signaling networks function somewhat redundantly at the genetic level [8]. Wnt and Netrin pathway loss of function can both mutually enhance and also suppress DTC migration defects caused by loss of function in the other pathway, revealing that each pathway contributes to both proper A/P and D/V DTC migration. Because of its apparent synergy with both Wnt and Netrin receptor genes, we next tested how the *mig-21(u787)* allele affected DTC migration in genetic contexts with combined Wnt and Netrin loss of function.

**Fig 5:**
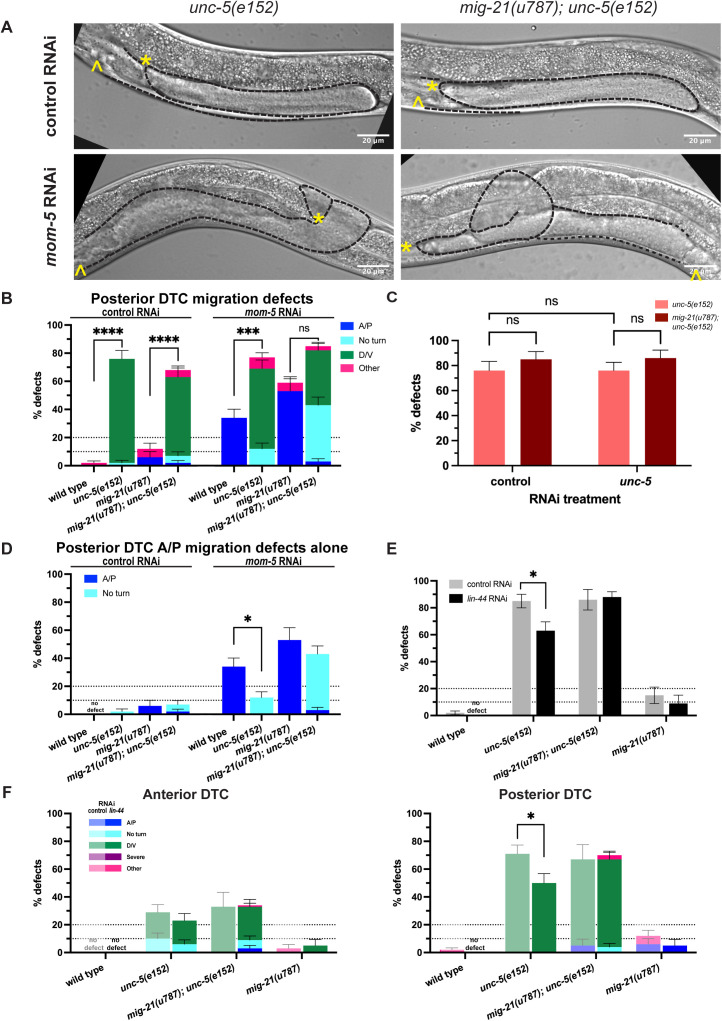
*mig-21* mediates Wnt-Netrin pathway crosstalk in the DTC. (A) Micrographs: DIC imaging of *C. elegans* hermaphrodites at the late larval L4 stage, comparing single mutants *unc-5(e152)* (left) and double *mig-21(u787); unc-5(e152)* (right) mutants under RNAi control L4440 empty vector and *mom-5* RNAi feeding treatment to assess DTC migration defect phenotypes. Images are Z-projections through 2-3 μm showing the distal gonad. Anterior left and ventral down. Black dashed lines outline gonads. Yellow asterisks mark DTC; yellow carets mark the vulval position. Scale bar: 20 μm. (B) Comparing the percentage of all classes of migration defects observed across different experimental groups in posterior gonad arms only. (C) All DTC migration defects across experimental groups comparing *unc-5(e152)* (salmon) and *mig-21(u787);unc-5(e152)* (cayenne) strains, under control RNAi L4440 empty vector and *unc-5* RNAi feeding treatments. (D) Comparing the percentage of only the classes containing anterior-posterior migration defects in posterior gonad arms shown in 5B. Anterior-posterior migration defects include “A/P” polarity reverse and “no turn” categories. Isolating these defects from the “D/V” and “other” classes makes it easier to see the significant suppression of *mom-5-*RNAi-induced A/P migration defects which is lost in *mig-21(u787);unc-5(e152)* double mutants on *mom-5* RNAi. (E) Comparing the percentage of all the DTC migration defects observed across different experimental groups under *lin-44* RNAi feeding treatment. The rate of *unc-5(e152)* migration defects (n=44/52) was significantly suppressed by *lin-44* RNAi (n=34/54). However, this suppression is lost in the *mig-21(u787); unc-5(e152)* genetic background. (F) Comparing the percentage of the DTC migration defect rates observed across different experimental groups in anterior (left) and posterior (right) arms for samples shown in 4E, with more specific defect categories and classifications. Suppression of *unc-5(e152)* D/V defects was not observed in anterior arms but was evident in posterior arms. Lighter one means under RNAi control L4440 empty vector, darker one means under *lin-44* RNAi. (B-F) All sample sizes refer to individual worms. (B, D-F) Datasets for N2 control strain on L4440 control and *lin-44* RNAi vector are from the same Wnt pathway RNAi experiment shown in Fig 2B. On the graphs, “no defect” means no defect observed in that group. Error bars represent the standard error of the sample proportion. Statistical analysis was performed using a pairwise proportion test, with p-values adjusted for multiple comparisons via the Benjamini-Hochberg procedure. Significant differences are indicated between groups where applicable. ****p < 0.0001; ***p < 0.001; **p < 0.01; *p < 0.05; no mark means the comparison was not statistically significant. The corresponding sample sizes and statistics are presented in Table AB–AJ in S1 File; additional details like raw data collections, calculated means, and SEM are presented in Sheets K-M in S2 File.

We first generated an *mig-21(u787); unc-5(e152)* double mutant. The *unc-5(e152)* allele has a premature stop codon truncating the intracellular domain of all isoforms [30–32]; that intracellular domain mediates the repulsion that brings about the first turn [35]. The *unc-5(e152)* mutant and the double mutant have no appreciable A/P migration defect beyond that of *mig-21(u787)* alone (Fig 5A and 5B). The *unc-5(e152)* mutant has an overall migration defect of 81% (n = 44/54, with a posterior bias, S2 Fig). This is a more penetrant defect than *unc-5* RNAi produces, and it is not enhanced by our *unc-5* RNAi (Fig 5C), suggesting that the *unc-5(e152)* mutant is a complete loss of function for *unc-5*-mediated regulation of DTC migration. We find that this migration defect rate is not significantly enhanced by *mig-21(u787)* (Fig 5B and 5C), providing further evidence that *unc-5* (via its C-terminal region) and *mig-21* act in the same DTC migration pathway and in the same direction. The reason for the discrepancy between the results for the *mig-21(u787)* allele combined with the *unc-5(e152)* mutant vs. *unc-5* RNAi is likely due to incomplete knockdown with *unc-5* RNAi [40].

With this double mutant, we interrogated the genetic interaction of *mom-5*/Frizzled and the *unc-5*/Netrin receptor. It is known that *mom-5* genetically represses *unc-5* (via positive regulation of Rac pathway components [17]). One key piece of evidence for this conclusion is that *mom-5* A/P migration defects are suppressed by *unc-5* loss of function [17], indicating that *mom-5* and *unc-5* affect A/P migration in opposite directions, and that *unc-5* is downstream of *mom-5* in the network. We hypothesized that this suppression would be maintained with *mig-21(u787)* in the genetic background, reflecting the redundancy of *mig-21* and *unc-5* (seen in Fig 5C).

Instead, we see something more complicated. Wild-type worms on *mom-5* RNAi have an A/P migration defect in the posterior DTC in a third of animals (Figs 2C and 5D n = 20/59, 33.9%). The *unc-5(e152)* mutant on *mom-5* RNAi shows suppression of A/P migration defects to 12.3% (n = 8/65, Fig 5B), as expected. However, the *mig-21(u787); unc-5(e152)* double mutant on *mom-5* RNAi does not significantly suppress the A/P migration defect (n = 31/72, 43%) relative to the *mig-21(u787)* mutant on *mom-5* RNAi alone (n = 17/32, 53% Fig 5D). These A/P migration defects are not made less frequent in *mig-21(u787); unc-5(e152)* but are converted to the “no turn” phenotype, which is considered to be a combination of a failed first turn and mispolarized second turn. Loss of function caused by *mig-21(u787)* simultaneously enhances A/P and D/V polarity defects caused by loss of function of *mom-5* and *unc-5* function, respectively. We conclude that suppression of *mom-5* RNAi A/P migration defects by loss of *unc-5* is *mig-21*-dependent.

We next tested a treatment by which loss of function of a Wnt signaling pathway member could suppress D/V migration defects caused by loss of *unc-5.* It had previously been shown that *lin-17*/Frizzled and *lin-44*/Wnt interact with *unc-5* in DTC migration [17]. That study focused on their redundant regulation of A/P polarity, but showed data that suggest that loss of function of those Wnt pathway members suppresses the D/V defects caused by *unc-5* loss of function. When we put the *unc-5(e152)* mutant on *lin-44*/Wnt RNAi, the *unc-5(e152)* D/V defect rate is suppressed from 79% (n = 41/52) to 63% (n = 34/54). This suppression is completely eliminated in a *mig-21(u787)* mutant background, with the D/V migration defect identical to the D/V defect rate in the *mig-21(u787); unc-5(e152)* mutant on RNAi vector control (n = 56/67, 83%, Fig 5E and 5F). We thus conclude that partial suppression of *unc-5(e152)* D/V migration defects by loss-of-function of a Wnt ligand is *mig-21*-dependent. Taken together, these results lead us to conclude that *mig-21* plays a key role in balancing the effects of Wnt and Netrin pathway signaling on DTC migration.

### *mig-21* is not required for cessation of DTC migration but does contribute to the wandering DTC migration caused by *vab-3* loss of function

**Fig 6:**
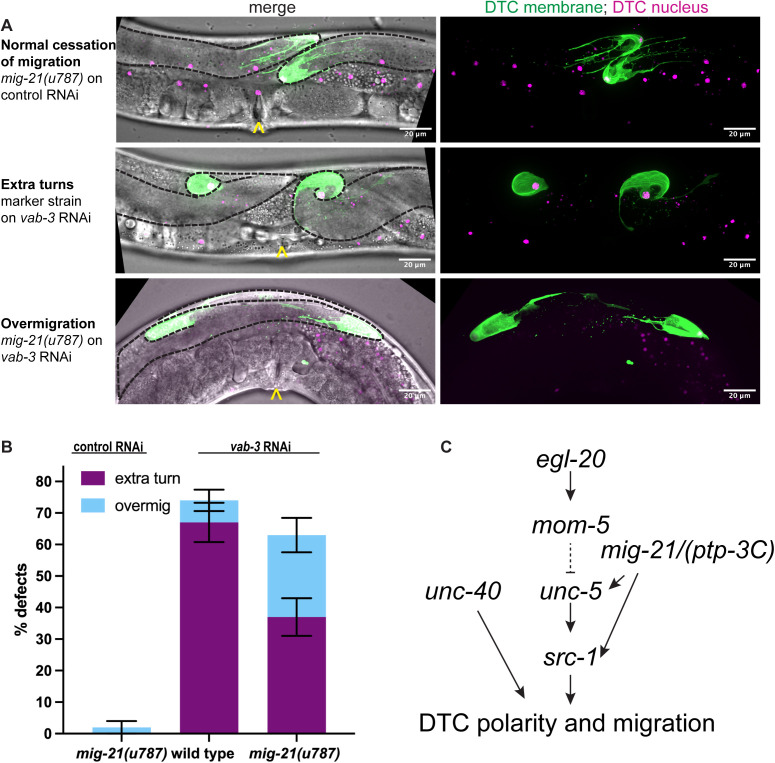
*mig-21* sensitizes adult DTCs to polarizing signals. (A) Micrographs: Confocal fluorescence imaging of *C. elegans* young adult hermaphrodites expressing (*lag-2p::mNG::PH; lag-2(bmd202[lag-2::P2A::H2B::mT2]*) without (middle) and with (top and bottom) *mig-21(u787)* under control (top) and *vab-3* RNAi (middle and bottom) feeding treatment to assess DTC migration cessation defect phenotypes. Images are Z-projections through thickness of the gonad required to capture the whole distal gonad. Black dashed lines outline gonads. Yellow carats mark proximal vulval position. Scale bar, 20 μm. (B) Comparing the percentage of two main DTC migrate cessation defect rates observed across different experimental groups under *vab-3* RNAi feeding treatment. An “extra turn” to “overmigration” defect shift was observed. A robust “extra turn” defect rate of control on *vab-3* RNAi (n=38/57) decreases with *mig-21(u787)* on *vab-3* RNAi (n=24/65), p < 0.01. However, migration cessation is not rescued; a significant increase in “overmigration” defects is observed between marked control (n=4/57) and *mig-21(u787)* on *vab-3* RNAi (n=17/65), p < 0.05. All sample sizes refer to individual worms. Error bars represent the standard error of the sample proportion. Statistical analysis was performed using a pairwise proportion test, with p-values adjusted for multiple comparisons via the Benjamini-Hochberg procedure. The corresponding sample sizes and statistics are presented in Table AK–AM in S1 File; additional details like raw data collections, calculated means, and SEM are presented in Sheet N in S2 File. (C) Proposed network of *mig-21* and its genetic interactors from this study, based on this and prior work. In our model, *mig-21* interacts genetically in the same pathway as *unc-5, src-1,* and *ptp-3C* and in parallel to the Wnt pathway and to *unc-40*, converging at or downstream of the levels of *unc-5* and *src-1* regulation. Dashed line indicates repression of *unc-5* by *mom-5*; this has been shown to involve the Rac-pathway members *mig-2, ced-10,* and *ced-12* [19]. Some of these factors are also shown to be epistatic to *src-1* [54], which itself is epistatic to *unc-5* [35], suggesting deeper complexity in their interactions. Since our results do not strongly support an interaction between *mig-21* and *ced-10* or *mig-2* (S1C–S1E Fig), we have chosen to leave *ced-10* and *mig-2* out of this pathway diagram.

We first began investigating *mig-21* because RNA-seq data shows that it is strongly and specifically expressed in adult DTCs, however, we went on to identify larval roles for *mig-21* in the DTC. We next asked if it interacts with a regulator of the adult DTC, *vab-3*/Pax6. This transcription factor is required for the cessation of DTC migration via the transcriptional switch in the alpha integrin subtype expressed by the DTC from *ina-1* to *pat-2* [33]. Loss-of-function of *vab-3* causes continued DTC migration in adulthood in which the DTC takes on a meandering or curling path [33]. Normally, DTC migration ends at the dorsal midbody, and *mig-21(u787)* mutants also cease migration in this position (Fig 6A). In otherwise-wild-type worms with DTC markers treated with *vab-3* RNAi, we see a high penetrance of overmigrated and curling gonad tips (Fig 6A). The defect rate was 75% n = 43/57), with 67% (n = 38/57) showing extra turns at the tip by 52–55 hrs post L1 arrest (Fig 6B). RNAi knockdown of *vab-3* combined with the *mig-21(u787)* allele displays a substantial shift from continued DTC migration with extra turns to DTC overmigration along the dorsal body wall along a straight path (Fig 6C and 6D). Cessation still fails (n = 48/65, 74%), but the DTC path stays straighter (n = 24/65, 37% have extra turns). If extra turns observed after *vab-3* RNAi treatment result from chaotic DTC polarization in response to signaling ligands in the adult, we interpret the suppression of those turns by *mig-21(u787)* to reflect a role for *mig-21* in the continued sensitization of the adult DTC to Wnt and Netrin signals, just as it is important for sensing and integrating this positional information in the larva.

## Conclusions and future directions

The nematode-specific *mig-21* gene encodes a thrombospondin repeat-containing protein that genetically interacts with Wnt, Netrin, and RPTP receptors during cell migration in both the Q neuroblast cells and–as we have now discovered–in the DTC. In both contexts, *mig-21* loss of function more strongly affects the cell that initially migrates to the posterior (this work and [12]), up the Wnt gradient. The molecular basis of these interactions is not known. Previous work [11] notes the thrombospondin domains shared by MIG-21 and UNC-5 could potentially mediate direct interactions between MIG-21 and UNC-40/DCC (as UNC-5 and UNC-40 were known to interact [41]; the genetic evidence, in that case, supports the parallel activity of the receptors [11,12]). In the case of the DTC, our results support *mig-21* acting in the same pathway as *unc-40, unc-5* and *src-1* in governing ventral repulsion and A/P polarity, and in parallel to *egl-20* and *mom-5*, converging on a common set of effectors to maintain a balance between Wnt and Netrin pathway regulation of DTC migration (Fig 6C).

MIG-21 and the UNC-40/DCC Netrin receptor are thought to regulate Wnt signal response in the Q neuroblast cells by restricting the direction of cell polarization [12]. It has subsequently been shown that *mig-21* together with *dpy-19* regulates UNC-40 subcellular localization to the leading edge of the polarizing Q neuroblast [42]. The proposed model by which MOM-5/Frizzled restricts UNC-5/Netrin receptor activity in the DTC proposed by [8] is strikingly similar–a limitation of the direction of cell polarization. Future studies of *mig-21* in the DTC will incorporate the ligands UNC-6/Netrin and UNC-129/TGF-β which regulate UNC-5 and UNC-40 signaling [43,44]). The UNC-6 ligand was proposed to impart directional information in such a way as to be important for UNC-5 function in the DTC [35], though recent work argues that the UNC-6 ligand is permissive rather than instructive of polarization in growth cones [29], and this may also be true of the DTC. In that case, polarization of the receptor could impart directional information that does not come from the ligand. In other systems, Frizzled protein itself is known to polarize in the cell membrane [45]. It is not yet known how these receptors localize during DTC migration.

What is currently known about DTC polarization instead concerns the localization of integrin-based extracellular matrix proteins INA-1 and PAT-3 (and their cytoskeletal linker TLN-1/talin) to the inside of a single U-turn [46], rather than two sequential 90 degree turns. In that framing, we would consider the D/V orientation and A/P orientation of the DTC as two directional vectors of a single 4-dimensional cell movement. Future studies will include assays on the direct effects of *mig-21(u787)* in combination with Wnt and Netrin pathway loss-of-function on polarity of these adhesion factors during turning.

One possibility is that MIG-21 affects UNC-5 localization in the DTC. This might explain the complicated genetic interactions among *mig-21, mom-5*, and *unc-5*, in which *mig-21* appears to act in the same direction as both *mom-5* and *unc-5*, even though *mom-5* itself negatively regulates *unc-5* in conferring A/P polarity to the DTC. Mislocalized UNC-5 protein could theoretically cause both loss of function phenotypes (not enough protein functioning in the right place) and overexpression phenotypes (too much protein functioning in the wrong place). Integrating MIG-21 into existing models of DTC migration will require further cell biological studies of receptor and effector protein polarization in the DTC, and in other cell types.

## Methods

*Sections of this text are adapted from K. Gordon lab publications* [16,47*] as they describe our standard laboratory practices.*

### Target gene selection

The target gene *mig-21* was selected from the single-cell transcriptional atlas of young adult *C. elegans* WormSeq.org app [9] designed by Ghaddar et al. [4]. The app offers various tools for gene expression analysis, including but not limited to identifying specific gene markers and assessing gene expression across cell types. In our study, we used the “top gene markers” function first to view the top 100 gene markers for the distal tip cell. This list was generated by the Monocle3 [13] “find markers” function, which “measures specificity using the Jensen-Shannon distance”. We first sorted the candidates by the highest “marker score” which yields genes that are both relatively specific and highly expressed, then we also sorted them by the “specificity” score as recommended by WormSeq.org app [9] We found that *mig-21* had the highest marker score and ranked third in specificity, and was the only gene present in the top ten of both sorts (Fig 1A).

### Strains

Some strains were provided by the CGC, which is funded by NIH Office of Research Infrastructure Programs (P40 OD010440). In strain descriptions, we designate linkage to a promoter with a p following the gene name and designate promoter fusions and in-frame fusions with a double semicolon (::). Some integrated strains (xxIs designation) may still contain for example the *unc-119* (ed4) mutation and/or the *unc-119* rescue transgene in their genetic background, but these are not listed in the strain description for the sake of concision, nor are most transgene 3’ UTR sequences. The wild-type strain N2 was used as a control. New strains produced for this study are:

KLG041 *cpIs122[lag-2p::mNeonGreen:: PLC*^*δ*^^*PH*^*] II; mig-21(u787) III; lag-2(bmd202[lag-2::P2A::H2B::mT2] ^lox511I^2xHA) V*
KLG042 *mig-21(u787) III; unc-5(e152) IV*
KLG048 *ptp-3(mu245), muls32 II; mig-21(u787) III*
KLG060 *cpIs121(lag-2p::mNG::PLC*^*δ*^^*PH*^*::F2A::rde-1) I*; *rrf-3(pk1426) II; mig-21(u787) III; rde-1(ne219) V*

### Worm rearing

*C. elegans* strains were maintained at 20°C on standard NGM media and fed *E. coli* OP50 for routine strain maintenance. All animals assessed were hermaphrodites, as males have nonmigratory DTCs. Worm populations were synchronized at L1 arrest for developmental staging by standard egg preps [48].

### Confocal imaging

All images were acquired on a Leica DMI8 with an xLIGHT V3 confocal spinning disk head (89 North) with a × 63 Plan-Apochromat (1.4 NA) objective and an ORCAFusion GenIII sCMOS camera (Hamamatsu Photonics) controlled by microManager [49]. mNG was excited with a 488 nm laser, and mT2 was excited by a 445 nm laser. Worms were mounted on 4% noble agar pads in 0.01 M sodium azide (VWR (Avantor) Catalog Number 97064–646) for live imaging.

### RNAi

*E. coli* HT115(DE3) containing the L4440 plasmid, either with or without a dsRNA trigger insert sourced from the Ahringer or Vidal Unique RNAi libraries, or our own clone in the case of *src-1* (see below) and *unc-5* [16], were cultured overnight from single colonies at 37°C with ampicillin (100 μg/mL, VWR (Avantor), Catalog no. 76204–346). Subsequently, dsRNA expression was induced with 1mM IPTG (Apex BioResearch Products, cat # 20–109) for one hour at 37°C, followed by plating 200 μl of the culture and incubating overnight at room temperature on prepared NGM plates with a 1:1 ratio (2.5 μL each) ampicillin and IPTG spread uniformly on the surface with a glass spreader. Worm populations were synchronized by bleaching according to a standard egg prep protocol [40], plated on NGM plates seeded with RNAi-expressing bacteria as arrested L1 larvae, and kept on RNAi until the time of imaging. RNAi treatment was conducted at 20°C. Replicates of N2 control strain on L4440 control RNAi vector (Figs 2, 3, 5 and S1) and *mig-21(u787)* strain on L4440 control RNAi vector (Figs 2 and 3) were run in parallel with each batch of experimental RNAis and these replicates were pooled in the resulting statistical analyses and the aforementioned figures (wild type N2 on control RNAi vector defects n = 102, *mig-21(u787)* on control RNAi vector n = 253).

RNAi against *ptp-3* is mediated by a 945 bp sequence of the fourth-to-last exon encoded by clone C09D8.1 in the Ahringer library [50] that is shared among all PTP-3 isoforms (S3C Fig). It does not have 100% similarity to any other genomic sequence greater than 20 bp long, making it unlikely that off-target RNAi knockdown explains why our results differ from results obtained with the *ptp-3(mu245)* mutant.

The RNAi clone targeting *src-1* was amplified from genomic DNA and cloned into the L4440 vector using Gibson cloning and the following primers sequences:

F 5’ TTGGGTACCGGGCCCCCCCTCGAGGATGAAGCAATGTGATCATCCGAATC
R 5’ GGCTGCAGGAATTCGATATCAAGCTTTAGGCACTTGGTGGCGCGTAATTC

DTC-specific RNAi strains NK2115 [51] and KLG060 were used to test cell-autonomous function of *mig-21* (S1B Fig) and its interactors (Fig 4). The NK2115 genetic background contains an *rrf-3(pk1426)* mutation that enhances RNAi, an *rde-1(ne219)* loss-of-function allele that prevents RNAi activity globally, and a membrane-targeted mNeonGreen cleaved from RDE-1 expressed by a rescue transgene driven by a *lag-2* promoter in the DTC: *cpIs121(lag-2p::mNG::PLC*^*δ*^^*PH*^*::F2A::rde-1)*. The strain KLG060 carries these genetic elements, and additionally carries the *mig-21(u787)* allele; it was generated by crossing a strain carrying this mutant allele to NK2115.

### Image analysis

Images were processed in FIJI (Version: 2.14.1/1.54f) [42]. Detailed descriptions of image analysis for different experiments are provided below.

### Measurements of DTC length and DTC nuclear location

The DTCs were identified in the fluorescence images (Fig 1E and 1F). The length of the DTC was determined as the distance from the gonad tip to the farthest point of the DTC edge. The location of the DTC nucleus was defined as the distance from the anatomical gonad tip to the center of the DTC nucleus. All measurements were obtained using the FIJI straight line tool.

### Staging and scoring of DTC migration defects

L4 (Figs 1–5 and S1–S3) or young adult (Fig 6) animals were scored for DTC migration defects based on gonad morphology. In Figs 1-5, we used the framework of [8] to categorize defects of A/P polarity, D/V polarity, and “no turn” defects in which D/V turning fails and A/P polarity is reversed. Some cases of A/P polarity defects result in DTC migration into the pharynx (anterior) or tail (posterior) regions, and others involve extra turns in which migration on the dorsal body wall started in these directions and subsequently reversed back towards the midbody. To these, we also add a “severe” category in which gonad outgrowth fails completely or the gonad forms a disorganized mass, and an “other” category, usually cases in which the last phase of gonad migration is not maintained along the dorsal body wall. In Fig 6, *vab-3* RNAi causes failure of migration cessation and perpetual migration, and we separate specimens into classic *vab-3* phenotypes in which the DTC makes extra turns and cases of “overmigration” in which the DTC maintains its path along the dorsal body wall but overshoots the midbody.

### Quantification and statistical analysis

Statistical tests, sample sizes, and p-values for some analyses are provided in the corresponding Results and Discussion text and all are provided in S1 File, with underlying data in the S2 File. Statistical analysis and multiple comparison corrections were performed using GraphPad Prism Version 10.4.0 (527) for macOS (GraphPad Software, Boston, MA, USA), and Rstudio version 2024.12.0 + 467 with the rstatix package [52]. The pairwise proportion test was used to compare proportions between experimental groups, and p-values were adjusted for multiple comparisons using the Benjamini-Hochberg procedure to control the False Discovery Rate (FDR) at 0.05. For histograms presented in the Figures, the standard error of the sample proportion was calculated with the $SE\hat{p}=\;\sqrt{\hat{p}(1-\hat{p})/n}$, where $\hat{p}$ is the proportion of specimens (worms or gonads) of the total observed *(n)* with the phenotype; error bars reflecting these standard errors (expressed as percentages) were added to each plot using GraphPad Prism. Asterisks appear on the graphs to reflect statistical significance in the observed proportions, and the p-values that correspond to those asterisks are explained in each figure legend. Further details of each analysis summarized in the figure legends are included in the referenced supplemental tables in File S1.

For the treatments for which the *mig-21(u787)* mutants significantly differed from the controls (as determined by the p-values given in the figures and supplemental tables in S1 File), we went on to test whether the genetic interaction was additive or super additive, and the results are reported in the relevant Results and Discussion text. We used a formula that predicts the additive effect of a double loss of function = (p1 + p2)-(p1*p2), with *p1* and *p2* being the proportion with the mutant phenotype in each single mutant/RNAi treatment [53]. We compared this predicted proportion to the observed proportion, assuming equal sample sizes, and tested for significance with a pairwise proportion test in R. If the difference was significant (and the observed rate was greater), we concluded that the genetic interaction is super additive. If the difference is not significant, we cannot rule out additivity. These are the p-values given in the text.

Unpaired Student’s two-tailed t-test was also performed to compare the means of DTC length and DTC nuclear location measurements, with p-values also calculated with GraphPad Prism.

## Supporting information

S1 Fig DTC migration defects in *mig-21(u787)* mutants expressing fluorescent markers under different treatment conditions. (A) DIC imaging of *mig-21(u787)* hermaphrodites at the late larval L4 stage without fluorescent markers (left) and with markers for the DTC membrane, *cpIs122[lag-2p::mNeonGreen:: PLC*^*δ*^^*PH*^], and a nuclear marker inserted at the endogenous *lag-2* locus *lag-2(bmd202[lag-2::P2A::H2B::mT2])* [16] merged with DIC (right). An additional defect of A/P polarity in DTC migration is sometimes observed and included in the “A/P” defect class in which the DTC exhibits polarity reversal upon meeting the dorsal body wall and later reverses its direction of migration 180 degrees. (B) Total DTC migration defects across experimental groups, including wild type, DTC-specific RNAi test strain NK2115 [51], and *mig-21(u787)* strains, under RNAi control L4440 empty vector (gray), and *mig-21* RNAi feeding treatments. (C) Micrographs: Confocal fluorescence imaging of the wild type and *mig-21(u787)* strains bearing a transgene that marks the membrane of the DTC, **cpIs122[lag-2p::mNeonGreen:: PLC*δ*PH]**, and a nuclear marker inserted at the *endogenous lag-2* *locus* *(lag-2::P2A::H2B::mT2).* Images are Z-projections through thickness of the gonad required to capture the whole distal gonad. S1A-B. Black dashed lines outline gonads. Yellow asterisks mark DTC; yellow carets mark the proximal vulval position. Scale bar: 20 μm. (D) DTC migration defects across experimental groups, including wild type N2 (gray) and *mig-21(u787)* (black) strains, under RNAi control L4440 empty vector, *mig-21* and *ced-10* RNAi feeding treatments. (E) DTC patch and bifurcated defects across experimental groups, including wild type N2 (left) and *mig-21(u787)* (right) strains, under RNAi control L4440 empty vector, *mig-21* and *ced-10* RNAi feeding treatments. (C-D) All sample sizes refer to individual worms. (D-E) Dataset for wild type strain on L4440 control RNAi vector is the same as shown in Figs 2B and 3B; these controls were pooled across replicates of all RNAi experiments. On the graphs, “no defect” means no defect observed in that group. Error bars represent the standard error of the sample proportion. Statistical analysis was performed using a pairwise proportion test, with p-values adjusted for multiple comparisons via the Benjamini-Hochberg procedure. Significant differences are indicated between groups where applicable. ****p < 0.0001; ***p < 0.001; **p < 0.01; *p < 0.05; no mark means the comparison was not statistically significant. The corresponding sample sizes and statistics are presented in Table AN–AR in S1 File; additional details like raw data collections, calculated means, and SEM are presented in Sheets O-Q in S2 File.
(TIF)

S2 Fig *unc-5(e152)* allele causes migration defects with a posterior bias that are not enhanced by *mig-21(u787)* and suppresses *mom-5* RNAi-mediated A/P migration defects. Control RNAi with empty vector (left grouping) and *mom-5* RNAi (right grouping). For each genotype (below), defects in the anterior DTC (left) and posterior DTC (right) are shown side by side. Posterior dataset is also shown in Fig 5B and analyzed in in Table AB in S1 File; Sheet R in S2 File.
(TIF)

S3 Fig Genetic interactions among *mig-21(u787)* and other migration factors. Micrographs: DIC imaging of *C. elegans* hermaphrodites at the late larval L4 stage, comparing *ptp-3(mu245)* (left) and *mig-21(u787); ptp-3(mu245)* (right). Images are Z-projections through 2–3 μm showing the distal gonad. Anterior left and ventral down. Black dashed lines outline gonads. Yellow asterisks mark DTC; yellow carets mark the proximal vulval position. Scale bar: 20 μm. (B) DTC migration defects across experimental groups, including *ptp-3(mu245)*, *mig-21(u787)*, and *mig-21(u787);ptp-3(mu245)* strains. (C) Genomic structure of *ptp-3* isoforms and RNAi targeting region (Wormbase [55]). Exons are depicted as magenta boxes; the connecting lines represent introns. The RNAi target region used in this study is indicated by the brown box at the bottom. (D) Micrographs: DIC imaging of *C. elegans* hermaphrodites at the late larval L4 stage, comparing *unc-5(e152)* (top) and *mig-21(u787); unc-5(e152)* (bottom) under *src-1* RNAi feeding treatment. Images are Z-projections through 2–3 μm showing the distal gonad. Anterior left and ventral down. Black dashed lines outline gonads. Yellow asterisks mark DTC; yellow carets mark the proximal vulval position. Scale bar: 20 μm. (E) All DTC migration defects across experimental groups comparing wild type*, mig-21(u787), unc-5(e152)* and *mig-21(u787);unc-5(e152)* strains, under control RNAi L4440 empty vector and *src-1* RNAi feeding treatments. (F-G) Comparing the percentage of the DTC migration defect rates observed across different experimental groups in anterior (left) and posterior (right) arms for samples shown in S3E, with more specific defect categories and classifications. (B, E-G) All sample sizes refer to individual worms. (B) Dataset for *mig-21(u787)* same as shown in Fig 1C. (E-G) Datasets for wild type strain on L4440 control RNAi vector and for wild type and *mig-21(u787)* on *src-1* RNAi are from the same experiment shown in Fig 3B and 3C. On the graphs, “no defect” means no defect observed in that group. Error bars represent the standard error of the sample proportion. Statistical analysis was performed using a pairwise proportion test, with p-values adjusted for multiple comparisons via the Benjamini-Hochberg procedure. Significant differences are indicated between groups where applicable. ****p < 0.0001; ***p < 0.001; **p < 0.01; *p < 0.05; no mark means the comparison was not statistically significant. The corresponding sample sizes and statistics are presented in Table AS–AX in S1 File; additional details like raw data collections, calculated means, and SEM are presented in Sheets S-T in S2 File.
(TIF)

S1 File Supporting data tables including sample sizes, statistical analyses, and tests of additivity relevant to the labeled corresponding experiments. Pairwise proportion tests were performed, with the Benjamini-Hochberg procedure used to adjust p-values for multiple comparisons.
(DOCX)

S2 File Raw data, calculated means, and standard errors of the mean (SEM) for all graphs presented in this study. Each sheet is labeled to correspond to the specific figure or experiment.
(XLSX)

## References

[1] Zhang S, Li X, Lin J, Lin Q, Wong K-C. Review of single-cell RNA-seq data clustering for cell-type identification and characterization. RNA. 2023;29(5):517–30. doi: 10.1261/rna.078965.121 36737104 PMC10158997

[2] Svensson V, da Veiga Beltrame E, Pachter L. A curated database reveals trends in single-cell transcriptomics. Database (Oxford). 2020;2020:baaa073. doi: 10.1093/database/baaa073 33247933 PMC7698659

[3] Plasschaert LW, Žilionis R, Choo-Wing R, Savova V, Knehr J, Roma G, et al. A single-cell atlas of the airway epithelium reveals the CFTR-rich pulmonary ionocyte. Nature. 2018;560(7718):377–81. doi: 10.1038/s41586-018-0394-6 30069046 PMC6108322

[4] Ghaddar A, Armingol E, Huynh C, Gevirtzman L, Lewis NE, Waterston R, et al. Whole-body gene expression atlas of an adult metazoan. Sci Adv. 2023;9(25):eadg0506. doi: 10.1126/sciadv.adg0506 37352352 PMC10289653

[5] Hubbard EJA, Greenstein D. TheCaenorhabditis elegans gonad: A test tube for cell and developmental biology. Dev Dyn. 2000;218(1):2–22. doi: 10.1002/(sici)1097-0177(200005)218:1<2::aid-dvdy2>3.0.co;2-w 10822256

[6] Gordon K. Recent Advances in the Genetic, Anatomical, and Environmental Regulation of the C. elegans Germ Line Progenitor Zone. J Dev Biol. 2020;8(3):14. doi: 10.3390/jdb8030014 32707774 PMC7559772

[7] Wong M-C, Schwarzbauer JE. Gonad morphogenesis and distal tip cell migration in the Caenorhabditis elegans hermaphrodite. Wiley Interdiscip Rev Dev Biol. 2012;1(4):519–31. doi: 10.1002/wdev.45 23559979 PMC3614366

[8] Levy-Strumpf N, Culotti JG. Netrins and Wnts function redundantly to regulate antero-posterior and dorso-ventral guidance in C. elegans. PLoS Genet. 2014;10(6):e1004381. doi: 10.1371/journal.pgen.1004381 24901837 PMC4046927

[9] Wormseq – Single-cell transcriptional atlas of adult C. elegans. https://wormseq.org/. Accessed 2025 January 21.

[10] Du H, Chalfie M. Genes regulating touch cell development in Caenorhabditis elegans. Genetics. 2001;158(1):197–207. doi: 10.1093/genetics/158.1.197 11333230 PMC1461620

[11] Middelkoop TC, Williams L, Yang P-T, Luchtenberg J, Betist MC, Ji N, et al. The thrombospondin repeat containing protein MIG-21 controls a left-right asymmetric Wnt signaling response in migrating C. elegans neuroblasts. Dev Biol. 2012;361(2):338–48. doi: 10.1016/j.ydbio.2011.10.029 22074987

[12] Sundararajan L, Lundquist EA. Transmembrane proteins UNC-40/DCC, PTP-3/LAR, and MIG-21 control anterior-posterior neuroblast migration with left-right functional asymmetry in Caenorhabditis elegans. Genetics. 2012;192(4):1373–88. doi: 10.1534/genetics.112.145706 23051647 PMC3512145

[13] Monocle. [cited 21 Jan 2025]. Available from: https://cole-trapnell-lab.github.io/monocle-release/monocle3/

[14] Patel DS, Garza-Garcia A, Nanji M, McElwee JJ, Ackerman D, Driscoll PC, et al. Clustering of genetically defined allele classes in the Caenorhabditis elegans DAF-2 insulin/IGF-1 receptor. Genetics. 2008;178(2):931–46. doi: 10.1534/genetics.107.070813 18245374 PMC2248335

[15] He F, Jacobson A. Nonsense-Mediated mRNA Decay: Degradation of Defective Transcripts Is Only Part of the Story. Annu Rev Genet. 2015;49:339–66. doi: 10.1146/annurev-genet-112414-054639 26436458 PMC4837945

[16] Singh N, Zhang P, Li KJ, Gordon KL. The Rac pathway prevents cell fragmentation in a nonprotrusively migrating leader cell during C. elegans gonad organogenesis. Curr Biol. 2024;34: 2387–402.e5. doi: 10.1016/j.cub.2024.04.073 38776905 PMC12013728

[17] Reddien PW, Horvitz HR. CED-2/CrkII and CED-10/Rac control phagocytosis and cell migration in Caenorhabditis elegans. Nat Cell Biol. 2000;2(3):131–6. doi: 10.1038/35004000 10707082

[18] Agarwal P, Berger S, Shemesh T, Zaidel-Bar R. Active nuclear positioning and actomyosin contractility maintain leader cell integrity during gonadogenesis. Curr Biol. 2024;34(11):2373-2386.e5. doi: 10.1016/j.cub.2024.03.049 38776903

[19] Levy-Strumpf N, Krizus M, Zheng H, Brown L, Culotti JG. The Wnt Frizzled Receptor MOM-5 Regulates the UNC-5 Netrin Receptor through Small GTPase-Dependent Signaling to Determine the Polarity of Migrating Cells. PLoS Genet. 2015;11(8):e1005446. doi: 10.1371/journal.pgen.1005446 26292279 PMC4546399

[20] Pani AM, Goldstein B. Direct visualization of a native Wnt in vivo reveals that a long-range Wnt gradient forms by extracellular dispersal. Elife. 2018;7:e38325. doi: 10.7554/eLife.38325 30106379 PMC6143344

[21] So S, Asakawa M, Sawa H. Distinct functions of three Wnt proteins control mirror-symmetric organogenesis in the C. elegans gonad. Elife. 2024;13:e103035. doi: 10.7554/eLife.103035 39485276 PMC11620738

[22] Cao J, Packer JS, Ramani V, Cusanovich DA, Huynh C, Daza R, et al. Comprehensive single-cell transcriptional profiling of a multicellular organism. Science. 2017;357(6352):661–7. doi: 10.1126/science.aam8940 28818938 PMC5894354

[23] Debant A, Serra-Pagès C, Seipel K, O’Brien S, Tang M, Park SH, et al. The multidomain protein Trio binds the LAR transmembrane tyrosine phosphatase, contains a protein kinase domain, and has separate rac-specific and rho-specific guanine nucleotide exchange factor domains. Proc Natl Acad Sci U S A. 1996;93(11):5466–71. doi: 10.1073/pnas.93.11.5466 8643598 PMC39269

[24] Gordon KL, Payne SG, Linden-High LM, Pani AM, Goldstein B, Hubbard EJA, et al. Ectopic Germ Cells Can Induce Niche-like Enwrapment by Neighboring Body Wall Muscle. Curr Biol. 2019;29(5):823-833.e5. doi: 10.1016/j.cub.2019.01.056 30799241 PMC6457669

[25] Tolkin T, Burnett J, Hubbard EJA. A role for organ level dynamics in morphogenesis of the C. elegans hermaphrodite distal tip cell. Development. 2024;151(19):dev203019. doi: 10.1242/dev.203019 39382030 PMC11488634

[26] Srinivasan S, Mahowald AP, Fuller MT. The receptor tyrosine phosphatase Lar regulates adhesion between Drosophila male germline stem cells and the niche. Development. 2012;139(8):1381–90. doi: 10.1242/dev.070052 22378638 PMC3308176

[27] Buettner FFR, Ashikov A, Tiemann B, Lehle L, Bakker H. C. elegans DPY-19 is a C-mannosyltransferase glycosylating thrombospondin repeats. Mol Cell. 2013;50(2):295–302. doi: 10.1016/j.molcel.2013.03.003 23562325

[28] Nichols EL, Lee J, Shen K. UNC-6/Netrin promotes both adhesion and directed growth within a single axon. eLife. 2025;13. doi: 10.7554/elife.100424.2 PMC11888602 40052533

[29] Hooper KM, Jain VD, Gormly CJ, Sanderson BJ, Lundquist EA. Short- and long-range roles of UNC-6/Netrin in dorsal-ventral axon guidance in vivo in Caenorhabditis elegans. PLoS Genet. 2025;21(1):e1011526. doi: 10.1371/journal.pgen.1011526 39823521 PMC11760026

[30] Chan SS, Zheng H, Su MW, Wilk R, Killeen MT, Hedgecock EM, et al. UNC-40, a C. elegans homolog of DCC (Deleted in Colorectal Cancer), is required in motile cells responding to UNC-6 netrin cues. Cell. 1996;87(2):187–95. doi: 10.1016/s0092-8674(00)81337-9 8861903

[31] Leung-Hagesteijn C, Spence AM, Stern BD, Zhou Y, Su MW, Hedgecock EM, et al. UNC-5, a transmembrane protein with immunoglobulin and thrombospondin type 1 domains, guides cell and pioneer axon migrations in C. elegans. Cell. 1992;71(2):289–99. doi: 10.1016/0092-8674(92)90357-i 1384987

[32] Hedgecock EM, Culotti JG, Hall DH. The unc-5, unc-6, and unc-40 genes guide circumferential migrations of pioneer axons and mesodermal cells on the epidermis in C. elegans. Neuron. 1990;4(1):61–85. doi: 10.1016/0896-6273(90)90444-k 2310575

[33] Meighan CM, Schwarzbauer JE. Control of C. elegans hermaphrodite gonad size and shape by vab-3/Pax6-mediated regulation of integrin receptors. Genes Dev. 2007;21(13):1615–20. doi: 10.1101/gad.1534807 17606640 PMC1899471

[34] Mahadik SS, Lundquist EA. A short isoform of the UNC-6/Netrin receptor UNC-5 is required for growth cone polarity and robust growth cone protrusion in Caenorhabditis elegans. Front Cell Dev Biol. 2023;11:1240994. doi: 10.3389/fcell.2023.1240994 37649551 PMC10464613

[35] Lee J, Li W, Guan K-L. SRC-1 mediates UNC-5 signaling in Caenorhabditis elegans. Mol Cell Biol. 2005;25(15):6485–95. doi: 10.1128/MCB.25.15.6485-6495.2005 16024786 PMC1190325

[36] Su M, Merz DC, Killeen MT, Zhou Y, Zheng H, Kramer JM, et al. Regulation of the UNC-5 netrin receptor initiates the first reorientation of migrating distal tip cells in Caenorhabditis elegans. Development. 2000;127(3):585–94. doi: 10.1242/dev.127.3.585 10631179

[37] Huang T-F, Cho C-Y, Cheng Y-T, Huang J-W, Wu Y-Z, Yeh AY-C, et al. BLMP-1/Blimp-1 regulates the spatiotemporal cell migration pattern in C. elegans. PLoS Genet. 2014;10(6):e1004428. doi: 10.1371/journal.pgen.1004428 24968003 PMC4072510

[38] Taylor SR, Santpere G, Weinreb A, Barrett A, Reilly MB, Xu C, et al. Molecular topography of an entire nervous system. Cell. 2021;184(16):4329-4347.e23. doi: 10.1016/j.cell.2021.06.023 34237253 PMC8710130

[39] Gao SM, Qi Y, Zhang Q, Guan Y, Lee Y-T, Ding L, et al. Aging atlas reveals cell-type-specific effects of pro-longevity strategies. Nat Aging. 2024;4(7):998–1013. doi: 10.1038/s43587-024-00631-1 38816550 PMC11257944

[40] Wang Z, Sherwood DR. Dissection of genetic pathways in C. elegans. Methods Cell Biol. 2011;106:113–57. doi: 10.1016/B978-0-12-544172-8.00005-0 22118276 PMC4116751

[41] Lim Y, Wadsworth WG. Identification of domains of netrin UNC-6 that mediate attractive and repulsive guidance and responses from cells and growth cones. J Neurosci. 2002;22(16):7080–7. doi: 10.1523/JNEUROSCI.22-16-07080.2002 12177204 PMC6757861

[42] Ebbing A, Middelkoop TC, Betist MC, Bodewes E, Korswagen HC. Partially overlapping guidance pathways focus the activity of UNC-40/DCC along the anteroposterior axis of polarizing neuroblasts. Development. 2019;146(18):dev180059. doi: 10.1242/dev.180059 31488562 PMC7376761

[43] Norris AD, Lundquist EA. UNC-6/netrin and its receptors UNC-5 and UNC-40/DCC modulate growth cone protrusion in vivo in C. elegans. Development. 2011;138(20):4433–42. doi: 10.1242/dev.068841 21880785 PMC3177313

[44] MacNeil LT, Hardy WR, Pawson T, Wrana JL, Culotti JG. UNC-129 regulates the balance between UNC-40 dependent and independent UNC-5 signaling pathways. Nat Neurosci. 2009;12(2):150–5. doi: 10.1038/nn.2256 19169249 PMC2745997

[45] Strutt DI. Asymmetric localization of frizzled and the establishment of cell polarity in the Drosophila wing. Mol Cell. 2001;7(2):367–75. doi: 10.1016/s1097-2765(01)00184-8 11239465

[46] Agarwal P, Shemesh T, Zaidel-Bar R. Directed cell invasion and asymmetric adhesion drive tissue elongation and turning in *C. elegans* gonad morphogenesis. Dev Cell. 2022;57: 2111–26.e6. doi: 10.1016/j.devcel.2022.08.003 36049484

[47] Li X, Singh N, Miller C, Washington I, Sosseh B, Gordon KL. The C. elegans gonadal sheath Sh1 cells extend asymmetrically over a differentiating germ cell population in the proliferative zone. Elife. 2022;11:e75497. doi: 10.7554/eLife.75497 36094368 PMC9467509

[48] Stiernagle T. Maintenance of C. elegans. WormBook. 2006;:1–11. doi: 10.1895/wormbook.1.101.1 18050451 PMC4781397

[49] Edelstein A, Amodaj N, Hoover K, Vale R, Stuurman N. Computer control of microscopes using µManager. Curr Protoc Mol Biol. 2010;92:Unit14.20. doi: 10.1002/0471142727.mb1420s92 20890901 PMC3065365

[50] Kamath RS, Ahringer J. Genome-wide RNAi screening in Caenorhabditis elegans. Methods. 2003;30(4):313–21. doi: 10.1016/s1046-2023(03)00050-1 12828945

[51] Linden LM, Gordon KL, Pani AM, Payne SG, Garde A, Burkholder D, et al. Identification of regulators of germ stem cell enwrapment by its niche in C. elegans. Dev Biol. 2017;429(1):271–84. doi: 10.1016/j.ydbio.2017.06.019 28648843 PMC5560089

[52] Kassambara A. rstatix: Pipe-Friendly Framework for Basic Statistical Tests. 2023. Available from: https://cran.r-project.org/web/packages/rstatix/index.html

[53] Mahadik SS, Burt EK, Lundquist EA. SRC-1 controls growth cone polarity and protrusion with the UNC-6/Netrin receptor UNC-5 in Caenorhabditis elegans. PLoS One. 2024;19(5):e0295701. doi: 10.1371/journal.pone.0295701 38771761 PMC11108135

[54] Itoh B, Hirose T, Takata N, Nishiwaki K, Koga M, Ohshima Y, et al. SRC-1, a non-receptor type of protein tyrosine kinase, controls the direction of cell and growth cone migration in C. elegans. Development. 2005;132(23):5161–72. doi: 10.1242/dev.02103 16251208

[55] Harris TW, Arnaboldi V, Cain S, Chan J, Chen WJ, Cho J, et al. WormBase: a modern Model Organism Information Resource. Nucleic Acids Res. 2020;48(D1):D762–7. doi: 10.1093/nar/gkz920 31642470 PMC7145598

