# Supplementary figures and images for: MIG-21 interacts with Wnt and Netrin signaling in gonad migration in C. elegans
Source: PLoS Genet. 2025 Sep 15;21(9):e1011866. doi: 10.1371/journal.pgen.1011866 (PMC12445740; doi:10.1371/journal.pgen.1011866)

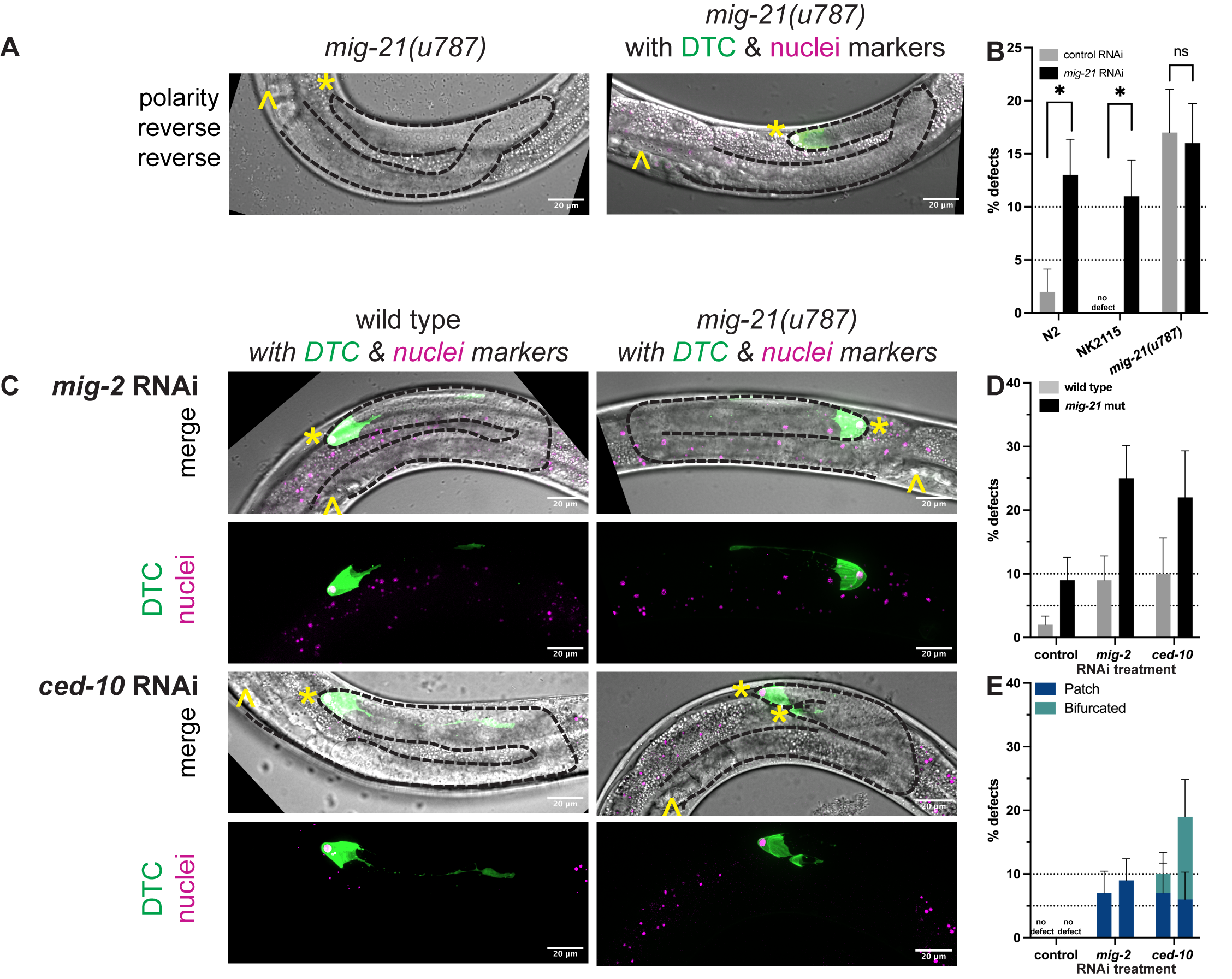

Supplement: S1 Fig — (A) DIC imaging of mig-21(u787) hermaphrodites at the late larval L4 stage without fluorescent markers (left) and with markers for the DTC membrane, cpIs122[lag-2p::mNeonGreen:: PLCδPH], and a nuclear marker inserted at the endogenous lag-2 locus lag-2(bmd202[lag-2::P2A::H2B::mT2]) [16] merged with DIC (right). An additional defect of A/P polarity in DTC migration is sometimes observed and included in the “A/P” defect class in which the DTC exhibits polarity reversal upon meeting the dorsal body wall and later reverses its direction of migration 180 degrees. (B) Total DTC migration defects across experimental groups, including wild type, DTC-specific RNAi test strain NK2115 [51], and mig-21(u787) strains, under RNAi control L4440 empty vector (gray), and mig-21 RNAi feeding treatments. (C) Micrographs: Confocal fluorescence imaging of the wild type and mig-21(u787) strains bearing a transgene that marks the membrane of the DTC, cpIs122[lag-2p::mNeonGreen:: PLCδPH], and a nuclear marker inserted at the endogenous lag-2 locus (lag-2::P2A::H2B::mT2). Images are Z-projections through thickness of the gonad required to capture the whole distal gonad. S1A-B. Black dashed lines outline gonads. Yellow asterisks mark DTC; yellow carets mark the proximal vulval position. Scale bar: 20 μm. (D) DTC migration defects across experimental groups, including wild type N2 (gray) and mig-21(u787) (black) strains, under RNAi control L4440 empty vector, mig-21 and ced-10 RNAi feeding treatments. (E) DTC patch and bifurcated defects across experimental groups, including wild type N2 (left) and mig-21(u787) (right) strains, under RNAi control L4440 empty vector, mig-21 and ced-10 RNAi feeding treatments. (C-D) All sample sizes refer to individual worms. (D-E) Dataset for wild type strain on L4440 control RNAi vector is the same as shown in Figs 2B and 3B; these controls were pooled across replicates of all RNAi experiments. On the graphs, “no defect” means no defect observed in that group [file pgen.1011866.s001.tif]

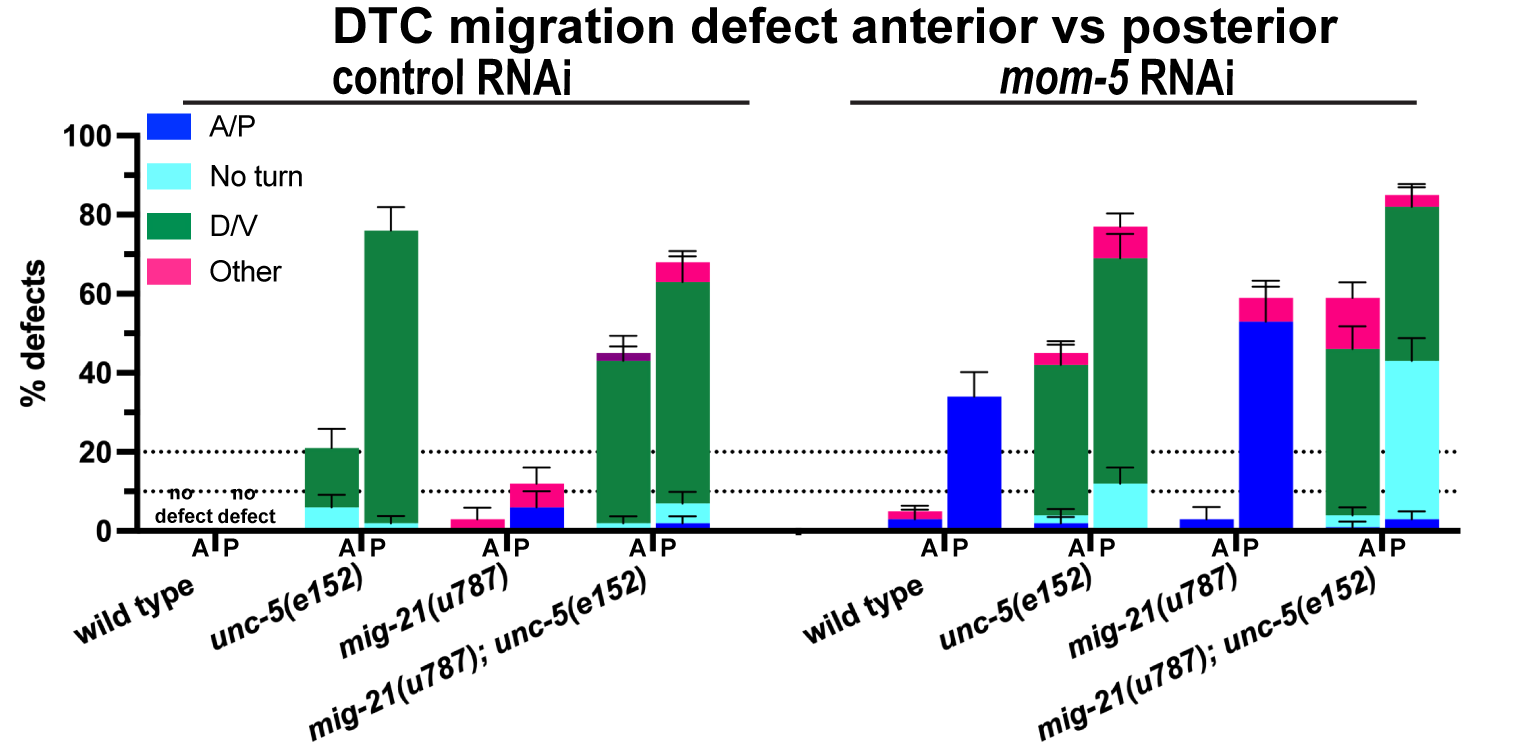

Supplement: S2 Fig — Control RNAi with empty vector (left grouping) and mom-5 RNAi (right grouping). For each genotype (below), defects in the anterior DTC (left) and posterior DTC (right) are shown side by side. Posterior dataset is also shown in Fig 5B and analyzed in in Table AB in S1 File; Sheet R in S2 File. (TIF) [file pgen.1011866.s002.tif]

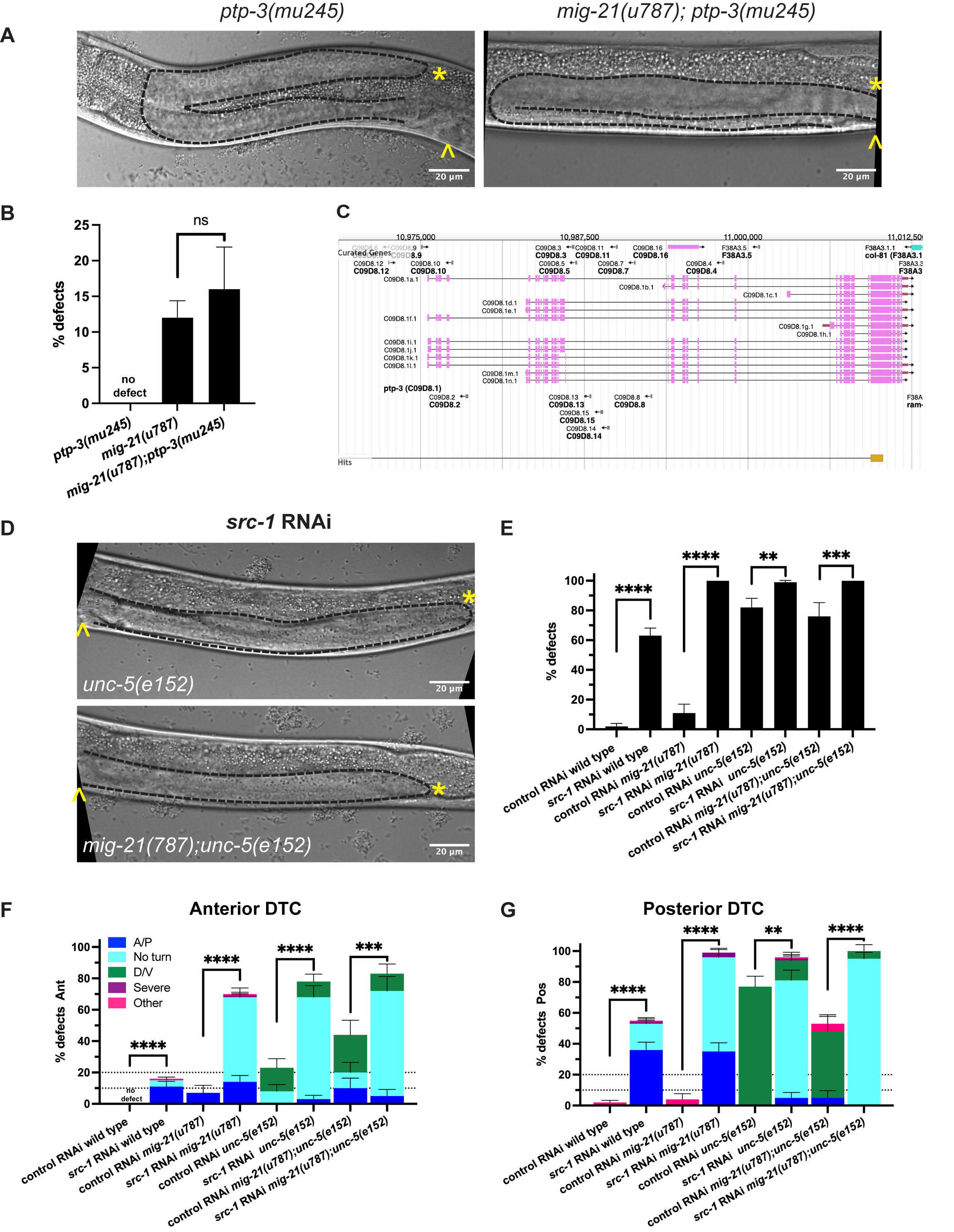

Supplement: S3 Fig — Micrographs: DIC imaging of C. elegans hermaphrodites at the late larval L4 stage, comparing ptp-3(mu245) (left) and mig-21(u787); ptp-3(mu245) (right). Images are Z-projections through 2–3 μm showing the distal gonad. Anterior left and ventral down. Black dashed lines outline gonads. Yellow asterisks mark DTC; yellow carets mark the proximal vulval position. Scale bar: 20 μm. (B) DTC migration defects across experimental groups, including ptp-3(mu245), mig-21(u787), and mig-21(u787);ptp-3(mu245) strains. (C) Genomic structure of ptp-3 isoforms and RNAi targeting region (Wormbase [55]). Exons are depicted as magenta boxes; the connecting lines represent introns. The RNAi target region used in this study is indicated by the brown box at the bottom. (D) Micrographs: DIC imaging of C. elegans hermaphrodites at the late larval L4 stage, comparing unc-5(e152) (top) and mig-21(u787); unc-5(e152) (bottom) under src-1 RNAi feeding treatment. Images are Z-projections through 2–3 μm showing the distal gonad. Anterior left and ventral down. Black dashed lines outline gonads. Yellow asterisks mark DTC; yellow carets mark the proximal vulval position. Scale bar: 20 μm. (E) All DTC migration defects across experimental groups comparing wild type, mig-21(u787), unc-5(e152) and mig-21(u787);unc-5(e152) strains, under control RNAi L4440 empty vector and src-1 RNAi feeding treatments. (F-G) Comparing the percentage of the DTC migration defect rates observed across different experimental groups in anterior (left) and posterior (right) arms for samples shown in S3E, with more specific defect categories and classifications. (B, E-G) All sample sizes refer to individual worms. (B) Dataset for mig-21(u787) same as shown in Fig 1C. (E-G) Datasets for wild type strain on L4440 control RNAi vector and for wild type and mig-21(u787) on src-1 RNAi are from the same experiment shown in Fig 3B and 3C. On the graphs, “no defect” means no defect observed in that group. Error bars represent the sta [file pgen.1011866.s003.tif]
