# Supplementary material for: MIG-21 interacts with Wnt and Netrin signaling in gonad migration in C. elegans
Source: PLoS Genet. 2025 Sep 15;21(9):e1011866. doi: 10.1371/journal.pgen.1011866 (PMC12445740; doi:10.1371/journal.pgen.1011866)
Supplement: S1 File — Pairwise proportion tests were performed, with the Benjamini-Hochberg procedure used to adjust p-values for multiple comparisons. (DOCX) [file pgen.1011866.s004.docx]

Table A: Sample Size of Fig. 1C

| Group | Total number | All defect |
| --- | --- | --- |
| N2 | 92 | 3 |
| *mig-21(u787)* | 189 | 23 |
| control with marker | 104 | 4 |
| *mig-21(u787)* with marker | 215 | 28 |

Table B: Statistical Results of Pairwise Proportion Tests for Fig. 1C

| Group 1 | Group 2 | p-value | p.adj | p.adj.signif |
| --- | --- | --- | --- | --- |
| N2 | control with marker | 1 | 1 | ns |
| N2 | *mig-21(u787)* | 0.0279 | 0.0478 | * |
| control with marker | *mig-21(u787)* | 0.0319 | 0.0478 | * |
| N2 | *mig-21(u787)*  with marker | 0.0167 | 0.0478 | * |
| control with marker | *mig-21(u787)*  with marker | 0.0183 | 0.0478 | * |
| *mig-21* | *mig-21(u787)*  with marker | 0.914 | 1 | ns |

Table C: Sample Size of Fig. 1D

| Group | Anterior defect | Posterior defect | A/P defect | D/V defect |
| --- | --- | --- | --- | --- |
| N2 | 1 | 2 | 1 | 0 |
| *mig21(u787)* | 7 | 18 | 19 | 1 |

Table D: Statistical Results of Pairwise Proportion Tests for Fig. 1D

| Group 1 | Group 2 | p-value | p.adj | p.adj.signif |
| --- | --- | --- | --- | --- |
| *mig-21(u787)* ant | *mig-21* (u787) pos | 0.0385 | 0.0385 | * |
| *mig-21(u787)* A/P def | *mig-21(u787)* D/V def | 0.0000938 | 0.0000938 | **** |

Table E: Sample Size of Fig. 2B-C

| Group | Total number | All defect | Anterior defect | Posterior defect |
| --- | --- | --- | --- | --- |
| control RNAi N2 | 102 | 2 | 0 | 2 |
| control RNAi *mig-21(u787)* | 253 | 23 | 9 | 17 |
| *mom-5* RNAi N2 | 202 | 56 | 16 | 40 |
| *mom-5* RNAi *mig-21(u787)* | 150 | 96 | 9 | 92 |
| *lin-17* RNAi N2 | 125 | 11 | 7 | 5 |
| *lin-17* RNAi *mig-21(u787)* | 138 | 25 | 17 | 10 |
| *egl-20* RNAi N2 | 110 | 3 | 3 | 3 |
| *egl-20* RNAi *mig-21(u787)* | 131 | 26 | 8 | 21 |
| *lin-44* RNAi N2 | 20 | 0 | 0 | 0 |
| *lin-44* RNAi *mig-21(u787)* | 22 | 2 | 1 | 1 |
| *mom-2* RNAi N2 | 58 | 3 | 0 | 3 |
| *mom-2* RNAi *mig-21(u787)* | 64 | 18 | 7 | 11 |
| *cwn-1* RNAi N2 | 23 | 0 | 1 | 0 |
| *cwn-1* RNAi *mig-21(u787)* | 39 | 6 | 1 | 5 |
| *cwn-2* RNAi N2 | 29 | 1 | 1 | 0 |
| *cwn-2* RNAi *mig-21(u787)* | 45 | 5 | 1 | 4 |

Table F: Statistical Results of Pairwise Proportion Tests for Fig. 2B

| Group 1 | Group 2 | p-value | p.adj | p.adj.signif |
| --- | --- | --- | --- | --- |
| control RNAi *mig-21* | *mom-5* RNAi *mig-21* | 6.00E-31 | 3.60E-30 | **** |
| *mom-5* RNAi control | *mom-5* RNAi *mig-21* | 2.29E-11 | 4.58E-11 | **** |
| control RNAi *mig-21* | *lin-17* RNAi *mig-21* | 0.0148 | 0.0444 | * |
| *lin-17* RNAi control | *lin-17* RNAi *mig-21* | 0.0439 | 0.0439 | * |
| control RNAi *mig-21* | *egl-20* RNAi *mig-21* | 4.60E-03 | 9.20E-03 | ** |
| *egl-20* RNAi control | *egl-20* RNAi *mig-21* | 1.09E-04 | 3.26E-04 | *** |
| control RNAi *mig-21* | *lin-44* RNAi *mig-21* | 1 | 1 | ns |
| *lin-44* RNAi control | *lin-44* RNAi *mig-21* | 0.512 | 0.767 | ns |
| control RNAi *mig-21* | *mom-2* RNAi *mig-21* | 0.00012 | 0.000361 | *** |
| *mom-2* RNAi control | *mom-2* RNAi *mig-21* | 0.00185 | 0.00369 | ** |
| control RNAi *mig-21* | *cwn-1* RNAi *mig-21* | 0.349 | 0.419 | ns |
| *cwn-1* RNAi control | *cwn-1* RNAi *mig-21* | 0.125 | 0.25 | ns |
| control RNAi *mig-21* | *cwn-2* RNAi *mig-21* | 0.88 | 1 | ns |
| *cwn-2* RNAi control | *cwn-2 RNAi* *mig-21* | 0.458 | 0.745 | ns |

Table G: Statistical Results of the Additive Test for Fig. 2B

|  | **Additive effect** | **Observed** | **n** | **Predicted additive for sample size (n)** | **Observed for sample size (n)** | **p-value** | **p.signif** |
| --- | --- | --- | --- | --- | --- | --- | --- |
| mom-5 | 34% | 64% | 150 | 52 | 96 | 6.85E-07 | **** |
| lin-17 | 17% | 18% | 138 | 24 | 25 | 1 | ns |
| egl-20 | 12% | 20% | 131 | 15 | 26 | 0.089 | ns |
| mom-2 | 14% | 28% | 64 | 9 | 18 | 0.083 | ns |

Table H: Statistical Results of Pairwise Proportion Test for Fig. 2C Anterior

| Group 1 | Group 2 | p-value | p.adj | p.adj.signif |
| --- | --- | --- | --- | --- |
| control RNAi *mig-21* | *mom-5* RNAi *mig-21* | 0.358 | 0.537 | ns |
| *mom-5* RNAi control | *mom-5* RNAi *mig-21* | 0.628 | 0.754 | ns |
| control RNAi *mig-21* | *lin-17* RNAi *mig-21* | 0.405 | 0.405 | ns |
| *lin-17* RNAi control | *lin-17* RNAi *mig-21* | 0.0939 | 0.141 | ns |
| control RNAi *mig-21* | *egl-20* RNAi *mig-21* | 0.412 | 0.412 | ns |
| *egl-20* RNAi control | *egl-20* RNAi *mig-21* | 0.346 | 0.412 | ns |
| control RNAi *mig-21* | *mom-2* RNAi *mig-21* | 0.832 | 0.832 | ns |
| *mom-2* RNAi control | *mom-2* RNAi *mig-21* | 0.0275 | 0.0438 | * |

Table I: Statistical Results of Pairwise Proportion Test for Fig. 2C Posterior

| Group 1 | Group 2 | p-value | p.adj | p.adj.signif |
| --- | --- | --- | --- | --- |
| control RNAi *mig-21* | *mom-5* RNAi *mig-21* | 1.09E-28 | 6.57E-28 | **** |
| *mom-5* RNAi control | *mom-5* RNAi *mig-21* | 4.23E-15 | 8.46E-15 | **** |
| control RNAi *mig-21* | *lin-17* RNAi *mig-21* | 0.662 | 0.662 | ns |
| *lin-17* RNAi control | *lin-17* RNAi *mig-21* | 0.386 | 0.579 | ns |
| control RNAi *mig-21* | *egl-20* RNAi *mig-21* | 0.0636 | 0.0763 | ns |
| *egl-20* RNAi control | *egl-20* RNAi *mig-21* | 0.00128 | 0.00385 | ** |
| control RNAi *mig-21* | *mom-2* RNAi *mig-21* | 0.1 | 0.15 | ns |
| *mom-2* RNAi control | *mom-2* RNAi *mig-21* | 0.0727 | 0.145 | ns |

Table J: Statistical Results of the Additive Test for Fig. 2C Posterior

|  | **Additive effect** | **Observed** | **n** | **Predicted additive for sample size (n)** | **Observed for sample size (n)** | **p-value** | **p.signif** |
| --- | --- | --- | --- | --- | --- | --- | --- |
| mom-5 | 34% | 64% | 150 | 52 | 96 | 6.85E-07 | **** |
| egl-20 | 12% | 20% | 131 | 15 | 26 | 0.089 | ns |

Table K: Sample Size of Fig. 3B-C

| Group | Total number | All defect | Anterior defect | Posterior defect | Anterior no turn | Posterior no turn |
| --- | --- | --- | --- | --- | --- | --- |
| control RNAi N2 | 102 | 2 | 0 | 2 | 0 | 0 |
| control RNAi *mig-21(u787)* | 253 | 23 | 9 | 17 | 0 | 0 |
| *ptp-3* RNAi N2 | 81 | 7 | 6 | 1 | 0 | 0 |
| *ptp-3* RNAi *mig-21(u787)* | 87 | 20 | 6 | 15 | 0 | 0 |
| *dpy-19* RNAi N2 | 53 | 3 | 2 | 1 | 0 | 0 |
| *dpy-19* RNAi *mig-21(u787)* | 79 | 9 | 6 | 5 | 0 | 0 |
| *unc-40* RNAi N2 | 93 | 16 | 10 | 15 | 0 | 0 |
| *unc-40* RNAi *mig-21(u787)* | 101 | 23 | 15 | 12 | 7 | 0 |
| *unc-5* RNAi N2 | 85 | 40 | 19 | 29 | 1 | 0 |
| *unc-5* RNAi *mig-21(u787)* | 103 | 78 | 46 | 54 | 30 | 20 |
| *src-1* RNAi N2 | 89 | 56 | 16 | 49 | 4 | 15 |
| *src-1* RNAi *mig-21(u787)* | 72 | 72 | 50 | 70 | 39 | 44 |

Table L: Statistical Results of Pairwise Proportion Tests for Fig. 3B

| Group 1 | Group 2 | p-value | p.adj | p.adj.signif |
| --- | --- | --- | --- | --- |
| control RNAi *mig-21* | *ptp-3* RNAi *mig-21* | 0.00149 | 0.00446 | ** |
| *ptp-3* RNAi control | *ptp-3* RNAi *mig-21* | 0.0204 | 0.0407 | * |
| control RNAi *mig-21* | *dpy-19* RNAi *mig-21* | 0.699 | 0.699 | ns |
| *dpy-19* RNAi control | *dpy-19* RNAi *mig-21* | 0.416 | 0.673 | ns |
| control RNAi *mig-21* | *unc-40* RNAi *mig-21* | 0.00103 | 0.00206 | ** |
| *unc-40* RNAi control | *unc-40* RNAi *mig-21* | 0.431 | 0.431 | ns |
| control RNAi *mig-21* | *unc-5* RNAi control | 5.99E-36 | 3.60E-35 | **** |
| *unc-5* RNAi control | *unc-5* RNAi *mig-21* | 9.80E-05 | 1.18E-04 | *** |
| control RNAi *mig-21* | *src-1* RNAi *mig-21* | 1.13E-49 | 6.80E-49 | **** |
| *src-1* RNAi control | *src-1* RNAi *mig-21* | 2.16E-08 | 2.60E-08 | **** |

Table M: Statistical Results of the Additive Test for Fig. 3B

|  | **Additive effect** | **Observed** | **n** | **Predicted additive for sample size (n)** | **Observed for sample size (n)** | **p-value** | **p.signif** |
| --- | --- | --- | --- | --- | --- | --- | --- |
| ptp-3 | 17% | 23% | 87 | 15 | 20 | 0.449 | ns |
| unc-5 | 52% | 76% | 103 | 53 | 78 | 0.0005 | *** |
| src-1 | 66% | 100% | 72 | 48 | 72 | 0.00000027 | **** |

Table N: Statistical Results of Pairwise Proportion Test for Fig. 3C Anterior

| Group 1 | Group 2 | p-value | p.adj | p.adj.signif |
| --- | --- | --- | --- | --- |
| control RNAi *mig-21* | *ptp-3* RNAi *mig-21* | 0.682 | 0.971 | ns |
| *ptp-3* RNAi control | *ptp-3* RNAi *mig-21* | 1 | 1 | ns |
| control RNAi *mig-21* | *unc-40* RNAi *mig-21* | 0.164 | 0.246 | ns |
| *unc-40* RNAi control | *unc-40* RNAi *mig-21* | 0.524 | 0.629 | ns |
| control RNAi *mig-21* | *unc-5* RNAi control | 4.33E-14 | 1.97E-13 | **** |
| *unc-5* RNAi control | *unc-5* RNAi *mig-21* | 2.31E-03 | 3.07E-03 | ** |
| control RNAi *mig-21* | *src-1* RNAi *mig-21* | 1.45E-26 | 8.71E-26 | **** |
| *src-1* RNAi control | *src-1* RNAi *mig-21* | 1.19E-10 | 2.38E-10 | **** |

Table O: Statistical Results of Pairwise Proportion Test for Fig. 3C Posterior

| Group 1 | Group 2 | p-value | p.adj | p.adj.signif |
| --- | --- | --- | --- | --- |
| control RNAi *mig-21* | *ptp-3* RNAi *mig-21* | 0.0595 | 0.0715 | ns |
| *ptp-3* RNAi control | *ptp-3* RNAi *mig-21* | 0.00108 | 0.00324 | ** |
| control RNAi *mig-21* | *unc-40* RNAi *mig-21* | 0.55 | 0.55 | ns |
| *unc-40* RNAi control | *unc-40* RNAi *mig-21* | 0.518 | 0.55 | ns |
| control RNAi *mig-21* | *unc-5* RNAi *mig-21* | 7.75E-19 | 4.65E-18 | **** |
| *unc-5* RNAi control | *unc-5* RNAi *mig-21* | 1.79E-02 | 2.14E-02 | * |
| control RNAi *mig-21* | *src-1* RNAi *mig-21* | 2.47E-47 | 1.48E-46 | **** |
| *src-1* RNAi control | *src-1* RNAi *mig-21* | 4.16E-09 | 4.99E-09 | **** |

Table P: Statistical Results of Pairwise Proportion Test for Fig. 3C, No turn phenotype (top Ant, bottom Pos)

| Group 1 | Group 2 | p-value | p.adj | p.adj.signif |
| --- | --- | --- | --- | --- |
| *unc-5* RNAi control | *unc-5* RNAi *mig-21* | 7.71E-07 | 1.29E-06 | **** |
| *unc-5* RNAi control | *unc-5* RNAi *mig-21* | 4.91E-05 | 4.91E-05 | **** |

Table Q: Statistical Results of the Additive Test for Fig. 3C Anterior

|  | **Additive effect** | **Observed** | **n** | **Predicted additive for sample size (n)** | **Observed for sample size (n)** | **p-value** | **p.signif** |
| --- | --- | --- | --- | --- | --- | --- | --- |
| unc-5 | 25% | 45% | 103 | 26 | 46 | 0.0055 | ** |
| src-1 | 21% | 69% | 72 | 16 | 50 | 3.41E-08 | **** |

Table R: Statistical Results of the Additive Test for Fig. 3C Posterior

|  | **Additive effect** | **Observed** | **n** | **Predicted additive for sample size (n)** | **Observed for sample size (n)** | **p-value** | **p.signif** |
| --- | --- | --- | --- | --- | --- | --- | --- |
| ptp-3 | 6% | 17% | 87 | 5 | 15 | 0.0324 | * |
| unc-5 | 39% | 52% | 103 | 40 | 54 | 0.069 | ns |
| src-1 | 58% | 97% | 72 | 42 | 70 | 6.23E-08 | **** |

Table S: Statistical Results of the Additive Test for A/P defects under *unc-40* RNAi

|  | **Additive effect** | **Observed** | **n** | **Predicted additive for sample size (n)** | **Observed for sample size (n)** | **p-value** | **p.signif** |
| --- | --- | --- | --- | --- | --- | --- | --- |
| unc-40 | 8% | 19% | 101 | 8 | 19 | 0.0387 | * |

Table T: Sample Size of Fig. 4B-C

| Group | Total number | All defect | Anterior defect | Posterior defect |
| --- | --- | --- | --- | --- |
| control RNAi N2 | 46 | 1 | 1 | 0 |
| control RNAi *mig-21(u787)* | 53 | 8 | 2 | 6 |
| control RNAi NK2115 | 84 | 1 | 1 | 0 |
| control RNAi KLG060 | 134 | 17 | 8 | 11 |
| *mom-5* RNAi N2 | 34 | 11 | 1 | 10 |
| *mom-5* RNAi *mig-21(u787)* | 42 | 29 | 2 | 26 |
| *mom-5* RNAi NK2115 | 73 | 55 | 10 | 54 |
| *mom-5* RNAi KLG060 | 107 | 101 | 16 | 98 |
| *unc-5* RNAi N2 | 62 | 20 | 6 | 14 |
| *unc-5* RNAi *mig-21(u787)* | 108 | 71 | 33 | 54 |
| *unc-5* RNAi NK2115 | 108 | 40 | 21 | 30 |
| *unc-5* RNAi KLG060 | 117 | 86 | 38 | 67 |

Table U: Statistical Results of Pairwise Proportion Tests for Fig. 3B *mom-5* section

| Group 1 | Group 2 | p-value | p.adj | p.adj.signif |
| --- | --- | --- | --- | --- |
| control RNAi N2 | control RNAi *mig-21* | 6.01E-02 | 6.01E-02 | ns |
| *mom-5* RNAi N2 | *mom-5* RNAi *mig-21* | 3.13E-03 | 4.70E-03 | ** |
| control RNAi NK2115 | control RNAi KLG060 | 5.99E-03 | 5.99E-03 | ** |
| *mom-5* RNAi NK2115 | *mom-5* RNAi KLG060 | 5.24E-04 | 6.28E-04 | *** |

Table V: Statistical Results of Pairwise Proportion Tests for Fig. 3B *unc-5* section

| Group 1 | Group 2 | p-value | p.adj | p.adj.signif |
| --- | --- | --- | --- | --- |
| control RNAi N2 | control RNAi *mig-21* | 6.01E-02 | 6.01E-02 | ns |
| *mom-5* RNAi N2 | *unc-5* RNAi *mig-21* | 5.05E-05 | 1.01E-04 | *** |
| control RNAi NK2115 | control RNAi KLG060 | 5.99E-03 | 5.99E-03 | ** |
| *unc-5* RNAi NK2115 | *unc-5* RNAi KLG060 | 7.83E-08 | 1.17E-07 | **** |

Table W: Statistical Results of the Additive Test for Fig. 4B DTC-specific RNAi groups

|  | **Additive effect** | **Observed** | **n** | **Predicted additive for sample size (n)** | **Observed for sample size (n)** | **p-value** | **p.signif** |
| --- | --- | --- | --- | --- | --- | --- | --- |
| mom-5 | 78% | 94% | 107 | 84 | 101 | 0.0014 | ** |
| unc-5 | 45% | 74% | 117 | 53 | 86 | 2.05E-05 | **** |

Table X: Statistical Results of Pairwise Proportion Tests for Fig. 4C Anterior *mom-5* section

| Group 1 | Group 2 | p-value | p.adj | p.adj.signif |
| --- | --- | --- | --- | --- |
| control RNAi NK2115 | control RNAi KLG060 | 0.169 | 0.202 | ns |
| *mom-5* RNAi NK2115 | *mom-5* RNAi KLG060 | 0.985 | 0.985 | ns |

Table Y: Statistical Results of Pairwise Proportion Tests for Fig. 4C Anterior *unc-5* section

| Group 1 | Group 2 | p-value | p.adj | p.adj.signif |
| --- | --- | --- | --- | --- |
| control RNAi NK2115 | control RNAi KLG060 | 0.169 | 0.169 | ns |
| *unc-5* RNAi NK2115 | *unc-5* RNAi KLG060 | 0.0385 | 0.0462 | * |

Table Z: Statistical Results of Pairwise Proportion Tests for Fig. 4C Posterior *mom-5* section

| Group 1 | Group 2 | p-value | p.adj | p.adj.signif |
| --- | --- | --- | --- | --- |
| control RNAi NK2115 | control RNAi KLG060 | 0.0175 | 0.0175 | * |
| *mom-5* RNAi NK2115 | *mom-5* RNAi KLG060 | 2.77E-03 | 3.32E-03 | ** |

Table AA: Statistical Results of Pairwise Proportion Tests for Fig. 4C Posterior *unc-5* section

| Group 1 | Group 2 | p-value | p.adj | p.adj.signif |
| --- | --- | --- | --- | --- |
| control RNAi NK2115 | control RNAi KLG060 | 0.0175 | 0.0175 | * |
| *unc-5* RNAi NK2115 | *unc-5* RNAi KLG060 | 1.51E-05 | 2.26E-05 | **** |

Table AB: Sample Size of Fig. 5B, D & Fig. S2

| Group | Total number | All defect | Anterior defect | Posterior defect | Posterior A/P | Posterior D/V |
| --- | --- | --- | --- | --- | --- | --- |
| control RNAi N2 | 102 | 2 | 0 | 2 | 0 | 0 |
| control RNAi *unc-5(e152)* | 54 | 44 | 11 | 41 | 1 | 41 |
| control RNAi *mig-21(u787)* | 34 | 5 | 1 | 4 | 2 | 0 |
| control RNAi *mig-21; unc-5(e152)* | 59 | 51 | 26 | 39 | 4 | 36 |
| *mom-5* RNAi N2 | 59 | 22 | 2 | 20 | 20 | 0 |
| *mom-5* RNAi *unc-5(e152)* | 65 | 56 | 29 | 46 | 8 | 45 |
| *mom-5* RNAi *mig-21(u787)* | 32 | 20 | 1 | 19 | 17 | 0 |
| *mom-5* RNAi *mig-21(u787);unc-5(e152)* | 72 | 67 | 37 | 55 | 31 | 57 |

Table AC: Statistical Results of Pairwise Proportion Tests for Fig. 5B

| Group 1 | Group 2 | p-value | p.adj | p.adj.signif |
| --- | --- | --- | --- | --- |
| control RNAi N2 | control RNAi *unc-5* | 5.03E-22 | 7.05E-21 | **** |
| control RNAi *mig-21* | control RNAi *mig-21;unc-5* | 1.26E-06 | 3.53E-06 | **** |
| *mom-5* RNAi N2 | *mom-5* RNAi *unc-5* | 8.52E-05 | 1.83E-04 | *** |
| *mom-5* RNAi *mig-21* | *mom-5* RNAi *mig-21;unc-5* | 0.125 | 0.185 | ns |

Table AD: Sample Size of Fig. 5C

| Group | Total number | All defect |
| --- | --- | --- |
| control RNAi *unc-5* | 34 | 26 |
| control RNAi *mig-21;unc-5* | 33 | 28 |
| *unc-5* RNAi *unc-5* | 42 | 32 |
| *unc-5* RNAi *mig-21;unc-5* | 29 | 25 |

Table AE: Statistical Results of Pairwise Proportion Tests for Fig. 5C

| Group 1 | Group 2 | p-value | p.adj | p.adj.signif |
| --- | --- | --- | --- | --- |
| control RNAi *unc-5* | control RNAi *mig-21;unc-5* | 0.577 | 0.865 | ns |
| control RNAi *unc-5* | *unc-5* RNAi *unc-5* | 1 | 1 | ns |
| control RNAi *mig-21;unc-5* | *unc-5* RNAi *unc-5* | 0.522 | 0.865 | ns |
| control RNAi *unc-5* | *unc-5* RNAi *mig-21;unc-5* | 0.51 | 0.865 | ns |
| control RNAi *mig-21;unc-5* | *unc-5* RNAi *mig-21;unc-5* | 1 | 1 | ns |
| *unc-5* RNAi *unc-5* | *unc-5* RNAi *mig-21;unc-5* | 0.46 | 0.865 | ns |

Table AF: Statistical Results of Pairwise Proportion Tests for Fig. 5D

| Group 1 | Group 2 | p-value | p.adj | p.adj.signif |
| --- | --- | --- | --- | --- |
| *mom-5* RNAi N2 | *mom-5* RNAi *unc-5* | 7.89E-03 | 1.38E-02 | * |
| *mom-5* RNAi *mig-21* | *mom-5* RNAi *mig-21;unc-5* | 4.62E-01 | 5.39E-01 | ns |

Table AG: Sample Size of Fig. 5E-F

| Group | Total number | All defect | Anterior defect | Posterior defect |
| --- | --- | --- | --- | --- |
| N2 control RNAi | 102 | 2 | 0 | 2 |
| *unc-5* control RNAi | 52 | 44 | 14 | 37 |
| *mig-21* control RNAi | 34 | 5 | 1 | 4 |
| *mig-21;unc-5* control RNAi | 21 | 18 | 7 | 14 |
| N2 on *lin-44* RNAi | 20 | 0 | 0 | 0 |
| *unc-5* on *lin-44* RNAi | 54 | 34 | 12 | 27 |
| *mig-21* on *lin-44* RNAi | 22 | 2 | 1 | 1 |
| *mig-21;unc-5* on *lin-44* RNAi | 67 | 59 | 23 | 47 |

Table AH: Statistical Results of Pairwise Proportion Tests for Fig. 5E

| Group 1 | Group 2 | p-value | p.adj | p.adj.signif |
| --- | --- | --- | --- | --- |
| control RNAi *unc-5* | *lin-44* RNAi *unc-5* | 2.10E-02 | 3.15E-02 | * |
| control RNAi *mig-21;unc-5* | *lin-44* RNAi *mig-21;unc-5* | 1.00E+00 | 1.00E+00 | ns |

Table AI: Statistical Results of Pairwise Proportion Tests for Fig. 5F Anterior

| Group 1 | Group 2 | p-value | p.adj | p.adj.signif |
| --- | --- | --- | --- | --- |
| control RNAi *unc-5* | *lin-44* RNAi *unc-5* | 0.736 | 0.736 | ns |
| control RNAi *mig-21;unc-5* | *lin-44* RNAi *mig-21;unc-5* | 1 | 1 | ns |
| *lin-44* RNAi *unc-5* | *lin-44* RNAi *mig-21;unc-5* | 0.208 | 0.208 | ns |

Table AJ: Statistical Results of Pairwise Proportion Tests for Fig. 5F Posterior

| Group 1 | Group 2 | p-value | p.adj | p.adj.signif |
| --- | --- | --- | --- | --- |
| control RNAi *unc-5* | *lin-44* RNAi *unc-5* | 0.0426 | 0.0426 | * |
| control RNAi *mig-21;unc-5* | *lin-44* RNAi *mig-21;unc-5* | 0.975 | 1 | ns |

Table AK: Sample Size of Fig. 6B

| Group | Total number | All defect | Extra turn | Overmigration |
| --- | --- | --- | --- | --- |
| control RNAi *mig-21(u787)* | 50 | 5 | 0 | 1 |
| *vab-3* RNAi N2 | 57 | 43 | 38 | 4 |
| *vab-3* RNAi *mig-21(u787)* | 65 | 48 | 24 | 17 |

Table AL: Statistical Results of Pairwise Proportion Tests for Fig. 6B Extra Turn

| Group 1 | Group 2 | p-value | p.adj | p.adj.signif |
| --- | --- | --- | --- | --- |
| *vab-3* RNAi control | *vab-3* RNAi *mig-21* | 1.95E-03 | 1.95E-03 | ** |

Table AM: Statistical Results of Pairwise Proportion Tests for Fig. 6B Overmigration

| Group 1 | Group 2 | p-value | p.adj | p.adj.signif |
| --- | --- | --- | --- | --- |
| *vab-3* RNAi control | *vab-3* RNAi *mig-21* | 0.0107 | 0.016 | * |

Table AN: Sample Size of Fig. S1B

| Group | Total number | DTC migration defect |
| --- | --- | --- |
| control RNAi N2 | 102 | 2 |
| *mig-21* RNAi N2 | 100 | 13 |
| control RNAi NK2115 | 56 | 0 |
| *mig-21* RNAi NK2115 | 83 | 9 |
| control RNAi *mig-21(u787)* | 84 | 14 |
| *mig-21* RNAi *mig-21(u787)* | 95 | 15 |

Table AO: Statistical Results of Pairwise Proportion Tests for Fig. S1B

| Group 1 | Group 2 | p-value | p.adj | p.adj.signif |
| --- | --- | --- | --- | --- |
| control RNAi N2 | *mig-21* RNAi N2 | 0.006 | 0.006 | * |
| control RNAi NK2115 | *mig-21* RNAi NK2115 | 0.028 | 0.028 | * |
| control RNAi *mig-21(u787)* | *mig-21* RNAi *mig-21(u787)* | 1 | 1 | ns |

Table AP: Sample Size of Fig. S1D-E

| Group | Total number | DTC migration defect |
| --- | --- | --- |
| control RNAi N2 | 102 | 2 |
| control RNAi *mig-21(u787)* | 65 | 6 |
| *mig-2* RNAi N2 | 56 | 5 |
| *mig-2* RNAi *mig-21(u787)* | 69 | 17 |
| *ced-10* RNAi N2 | 29 | 3 |
| *ced-10* RNAi *mig-21(u787)* | 32 | 7 |

Table AQ: Statistical Results of Pairwise Proportion Tests for Fig. S1D

| Group 1 | Group 2 | p-value | p.adj | p.adj.signif |
| --- | --- | --- | --- | --- |
| control RNAi *mig-21* | *mig-2* RNAi *mig-21* | 0.0011 | 0.00329 | ** |
| *mig-2* RNAi control | *mig-2* RNAi *mig-21* | 0.0397 | 0.0595 | ns |
| control RNAi *mig-21* | *ced-10* RNAi *mig-21* | 0.0556 | 0.111 | ns |
| *ced-10* RNAi control | *ced-10* RNAi *mig-21* | 0.385 | 0.462 | ns |

Table AR: Statistical Results of Pairwise Proportion Tests for Fig. S1E

| Group 1 | Group 2 | p-value | p.adj | p.adj.signif |
| --- | --- | --- | --- | --- |
| *mig-2* RNAi control | *mig-2* RNAi *mig-21* | 1 | 1 | ns |
| *ced-10* RNAi *control* | *ced-10* RNAi *mig-21* | 0.573 | 0.573 | ns |

Table AS: Sample Size of Fig. S3B

| Group | Total number | DTC migration defect |
| --- | --- | --- |
| *ptp-3(mu245)* | 25 | 0 |
| *mig-21(u787)* | 189 | 23 |
| *mig-21(u787);ptp-3(mu245)* | 38 | 6 |

Table AT: Statistical Results of Pairwise Proportion Tests for Fig. S3B

| Group 1 | Group 2 | p-value | p.adj | p.adj.signif |
| --- | --- | --- | --- | --- |
| *ptp-3(mu245)* | *mig-21(u787)* | 0.133 | 0.199 | ns |
| *ptp-3(mu245)* | *mig-21(u787);ptp-3(mu245)* | 0.0989 | 0.199 | ns |
| *mig-21(u787)* | *mig-21(u787);ptp-3(mu245)* | 0.731 | 0.731 | ns |

Table AU: Sample Size of Fig. S3E-G

| Group | Total number | All defect | Anterior defect | Posterior defect |
| --- | --- | --- | --- | --- |
| control RNAi N2 | 102 | 2 | 0 | 2 |
| *src-1* RNAi N2 | 89 | 56 | 16 | 49 |
| control RNAi *mig-21(u787)* | 28 | 3 | 2 | 1 |
| src-1 RNAi *mig-21(u787)* | 72 | 72 | 50 | 70 |
| control RNAi *unc-5(e152)* | 39 | 32 | 9 | 30 |
| *src-1* RNAi *unc-5(152)* | 21 | 16 | 9 | 11 |
| control RNAi  *mig-21(u787);unc-5(e152)* | 78 | 77 | 62 | 76 |
| *src-1* RNAi  *mig-21(u787);unc-5(e152)* | 64 | 64 | 54 | 64 |

Table AV: Statistical Results of Pairwise Proportion Tests for Fig. S3E

| Group 1 | Group 2 | p-value | p.adj | p.adj.signif |
| --- | --- | --- | --- | --- |
| control RNAi N2 | *src-1* RNAi N2 | 2.66E-19 | 1.03E-18 | **** |
| control RNAi *mig-21(u787)* | *src-1* RNAi *mig-21(u787)* | 2.24E-19 | 1.01E-18 | **** |
| control RNAi *unc-5(e152)* | *src-1* RNAi *unc-5(e152)* | 2.90E-03 | 3.72E-03 | ** |
| control RNAi *mig-21(u787);unc-5(e152)* | *src-1* RNAi *mig-21(u787);unc-5(e152)* | 4.84E-04 | 7.69E-04 | *** |

Table AW: Statistical Results of Pairwise Proportion Tests for Fig. S3F

| Group 1 | Group 2 | p-value | p.adj | p.adj.signif |
| --- | --- | --- | --- | --- |
| control RNAi N2 | *src-1* RNAi N2 | 2.53E-05 | 4.73E-05 | **** |
| control RNAi *mig-21(u787)* | *src-1* RNAi *mig-21(u787)* | 7.61E-08 | 1.77E-07 | **** |
| control RNAi *unc-5(e152)* | *src-1* RNAi *unc-5(e152)* | 1.29E-08 | 3.27E-08 | **** |
| control RNAi *mig-21(u787);unc-5(e152)* | *src-1* RNAi *mig-21(u787);unc-5(e152)* | 4.97E-04 | 8.70E-04 | *** |

Table AX: Statistical Results of Pairwise Proportion Tests for Fig. S3G

| Group 1 | Group 2 | p-value | p.adj | p.adj.signif |
| --- | --- | --- | --- | --- |
| control RNAi N2 | *src-1* RNAi N2 | 5.06E-16 | 1.77E-15 | **** |
| control RNAi *mig-21(u787)* | *src-1* RNAi *mig-21(u787)* | 1.86E-19 | 7.43E-19 | **** |
| control RNAi *unc-5(e152)* | *src-1* RNAi *unc-5(e152)* | 1.16E-03 | 1.63E-03 | ** |
| control RNAi *mig-21(u787);unc-5(e152)* | *src-1* RNAi *mig-21(u787);unc-5(e152)* | 4.09E-08 | 8.19E-08 | **** |
